# Tracking live-cell single-molecule dynamics enables measurements of heterochromatin-associated protein–protein interactions

**DOI:** 10.1093/nar/gkae692

**Published:** 2024-08-15

**Authors:** Ziyuan Chen, Melissa Seman, Yekaterina Fyodorova, Ali Farhat, Amanda Ames, Alexander Levashkevich, Saikat Biswas, Fengting Huang, Lydia Freddolino, Julie S Biteen, Kaushik Ragunathan

**Affiliations:** Department of Biophysics, University of Michigan, Ann Arbor, MI 48109, USA; Department of Biology, Brandeis University, Waltham, MA 02451, USA; Department of Chemistry, University of Michigan, Ann Arbor, MI 48109, USA; Department of Computational Medicine and Bioinformatics, University of Michigan Medical School, Ann Arbor, MI 48109, USA; Department of Biology, Brandeis University, Waltham, MA 02451, USA; Department of Biology, Brandeis University, Waltham, MA 02451, USA; Department of Biological Chemistry, University of Michigan Medical School, Ann Arbor, MI 48109, USA; Department of Biology, Brandeis University, Waltham, MA 02451, USA; Department of Computational Medicine and Bioinformatics, University of Michigan Medical School, Ann Arbor, MI 48109, USA; Department of Biological Chemistry, University of Michigan Medical School, Ann Arbor, MI 48109, USA; Department of Biophysics, University of Michigan, Ann Arbor, MI 48109, USA; Department of Chemistry, University of Michigan, Ann Arbor, MI 48109, USA; Department of Biology, Brandeis University, Waltham, MA 02451, USA

## Abstract

Visualizing and measuring molecular-scale interactions in living cells represents a major challenge, but recent advances in single-molecule super-resolution microscopy are bringing us closer to achieving this goal. Single-molecule super-resolution microscopy enables high-resolution and sensitive imaging of the positions and movement of molecules in living cells. HP1 proteins are important regulators of gene expression because they selectively bind and recognize H3K9 methylated (H3K9me) histones to form heterochromatin-associated protein complexes that silence gene expression, but several important mechanistic details of this process remain unexplored. Here, we extended live-cell single-molecule tracking studies in fission yeast to determine how HP1 proteins interact with their binding partners in the nucleus. We measured how genetic perturbations that affect H3K9me alter the diffusive properties of HP1 proteins and their binding partners, and we inferred their most likely interaction sites. Our results demonstrate that H3K9 methylation spatially restricts HP1 proteins and their interactors, thereby promoting ternary complex formation on chromatin while simultaneously suppressing off-chromatin binding. As opposed to being an inert platform to direct HP1 binding, our studies propose a novel function for H3K9me in promoting ternary complex formation by enhancing the specificity and stimulating the assembly of HP1–protein complexes in living cells.

## Graphical Abstract

**Graphical Abstract:**
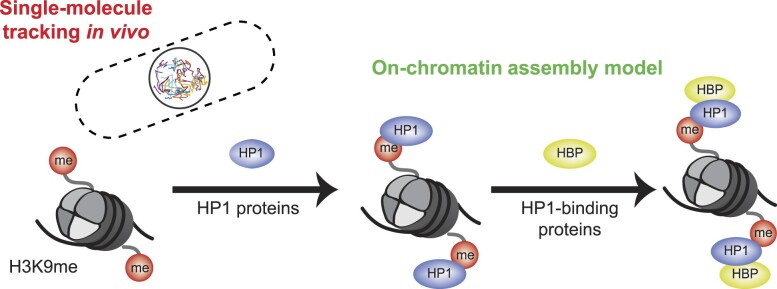

## Introduction

Genetically identical cells exhibit different phenotypes due to the covalent modifications of DNA packaging proteins called histones (1). These modifications give rise to heritable changes in gene expression, dividing the genome into two physically distinct compartments based on their intrinsic expression patterns: active euchromatin and silent heterochromatin (1). Certain histone modifications, like H3 lysine 9 methylation (H3K9me), are enriched within heterochromatin (2–6). Proper heterochromatin assembly is important for maintaining genome integrity, silencing repetitive DNA sequences, and preserving cell identity (5,7). H3K9me acts as a scaffold that promotes the binding and recruitment of a conserved family of proteins called HP1. These proteins bind and dissociate rapidly from chromatin on the milliseconds to seconds timescale (8–11). HP1 proteins orchestrate the dynamic assembly of large multi-protein complexes that alter chromatin structure, genome organization, and transcription (2,12–18). Paradoxically, HP1 proteins simultaneously interact with and recruit a host of silencing and anti-silencing factors to sites of heterochromatin formation (19). Any imbalance in this recruitment process could lead to improper heterochromatin assembly and jeopardize the integrity of gene silencing (20). Furthermore, since different HP1 complexes bind to H3K9me, we do not understand how their interactions with a shared chromatin substrate lead to productive heterochromatin assembly as opposed to competition.

Current methods for detecting protein–protein interactions cannot measure real-time dynamic processes in living cells. Immunoprecipitation followed by mass spectrometry (IP-MS) detects protein–protein interactions, yet the outcome of such measurements can depend on factors such as lysis conditions, salt concentrations, and protein abundance. As a result, these assays may not represent the full range of interactions that occur between proteins in living cells. Detecting protein–protein interactions after lysing cells also extricates proteins from their native, crowded chromatin environment, so the *in vitro* properties of chromatin-associated factors can exhibit inconsistencies relative to their behavior in cells (21). Attempts to bridge the gap between *in vitro* and *in vivo* observations with techniques such as Förster resonance energy transfer (FRET) and two-color imaging rely on protein–protein interactions that are infrequent and transient given their dynamic properties. Additionally, FRET poses methodological challenges due to its limited working distance (<10 nm) and rare, spontaneous interactions between labeled molecules (22).

Live-cell super-resolution microscopy and single-molecule tracking are powerful tools for studying protein dynamics on the nanometer scale *in vivo* (23–25). Using photoactivatable fluorescent protein tags, we can track individual molecules to access the millisecond timescale interactions of histone modifiers with their chromatin substrates in living cells (21,26,27). The dynamics of these proteins in the cell nucleus represent the full range of possible interactions: protein diffusion is slowed by transient interactions and significantly reduced by binding to modified histones. Hence, by imaging individual molecules in their native chromatin context we can analyze complex trajectories that arise from protein interactions within a heterogeneous cellular environment (28,29). This framework enables us to connect our biophysical measurements (a set of mobility states, each with an average diffusion coefficient) to protein–protein and protein-substrate interactions (a function of binding affinities) in living cells (21).

The fission yeast, *Schizosaccharomyces pombe*, has two HP1 orthologs, Swi6 and Chp2, which bind to H3K9me and promote transcriptional gene silencing (15,30,31). HP1 proteins have two conserved domains; a chromodomain that binds H3K9me with high specificity and a chromoshadow domain that promotes dimerization and protein–protein interactions. These domains are separated by an unstructured intrinsically disordered hinge region that is potentially involved in binding non-specifically to DNA or RNA (32). Despite their structural similarity and shared evolutionary origin, Swi6 and Chp2 are expressed at very different levels in the cell and have distinct roles in heterochromatin formation (20,33). *In vitro*, Swi6 and Chp2 have similar tendencies to form dimers and oligomers (14,20,34,35), but Swi6 potentially binds to nucleosomes approximately 3-fold more strongly than Chp2 (34).

**Figure 1.**
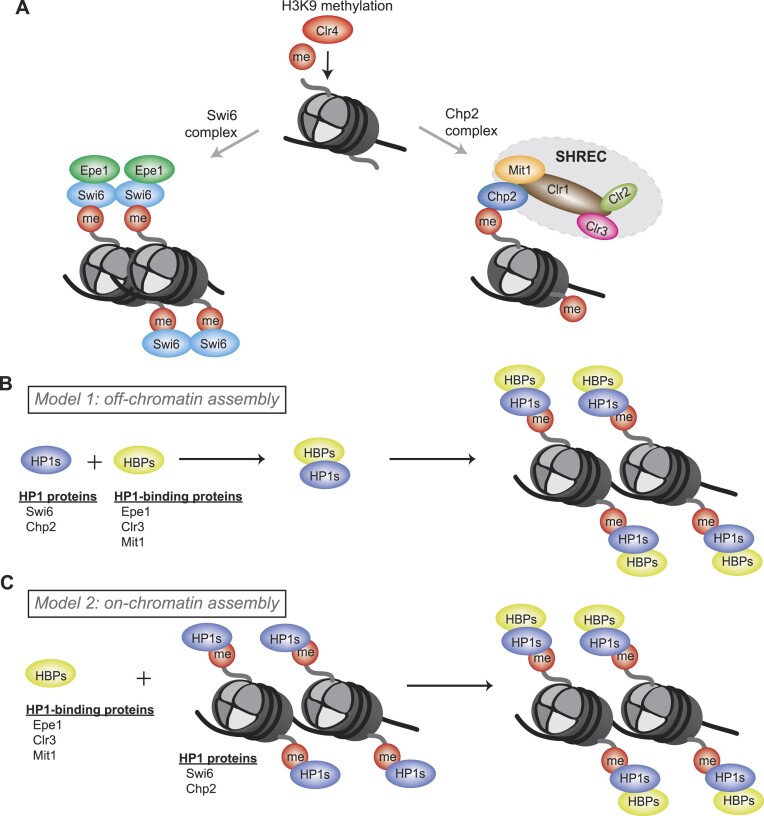
Models of HP1-associated complex assembly. (**A**) Swi6 and Chp2 interact with different sets of heterochromatin-associated factors at sites of H3K9me. (**B**) Model 1: off-chromatin assembly model. HP1 proteins and their binding proteins bind off-chromatin and then survey the genome to find H3K9me sites. (**C**) Model 2: on-chromatin assembly model. HP1 proteins form complexes with their binding partners exclusively at sites of H3K9me.

Swi6 and Chp2 also preferentially interact with different sets of heterochromatin-associated factors. Epe1, a putative H3K9 demethylase and the primary anti-silencing factor in fission yeast cells (36) interacts with Swi6 both *in vitro* and *in vivo* (37,38) (Figure 1A). On the other hand, the *S. pombe* SHREC complex, which consists of two major chromatin-modifying enzymes, Clr3 and Mit1, preferentially forms complexes with Chp2 (36,39–41) (Figure 1A). Two potential models explain how HP1 proteins form specific complexes in the cell: The first possibility is that Swi6 and Chp2 bind to their respective partner proteins (Epe1 with Swi6 or Mit1 and Clr3 with Chp2) off-chromatin, forming binary complexes to survey the genome and ultimately localize at sites of H3K9me (Figure 1B). However, cells lacking H3K9me (*clr4Δ)* exhibit a precipitous loss of HP1-mediated protein interactions, suggesting that these complexes are unstable when not bound to chromatin (19). An alternative model is that HP1 proteins form complexes with their binding partners exclusively at sites of H3K9me rather than off-chromatin. This would imply that H3K9me chromatin directly participates in the formation of stable ternary complexes in living cells (Figure 1C).

To differentiate between these two models, we used live-cell single-molecule fluorescence microscopy to measure the dynamics and interactions of the HP1 proteins Swi6 and Chp2 and their primary interacting partners. Our results suggest that HP1 proteins form complexes with their binding partners preferentially at sites of H3K9me, indicating that H3K9me chromatin promotes a high-affinity binding mode which in turn suppresses off-chromatin binding by spatially sequestering molecules at their sites of action. Our data lead us to suggest a novel function for H3K9me in promoting HP1-dependent ternary complex assembly in living cells.

## Materials and methods

### Plasmids

All fluorescently tagged proteins were made with Gibson cloning. Strains with *nmt* promoters were constructed by modifying existing pDual plasmids (42).

### Strains

Most strains were constructed using a PCR-based gene-targeting approach (43). All strains with a PAmCherry fluorescent tag were made by constructing pDual vectors, containing the specified *nmt* promoter and the indicated protein (42). The plasmid was digested with the restriction enzyme, NotI, and the digested plasmid was transformed into *leu1-32* strains to select for growth on EMM-leu media (minimal media lacking leucine) as the pDual vector restores a functional *leu1*+ gene. Epe1-PAmCherry was made by using long oligos to tag Epe1 at its C-terminus. Deletions were made by a PCR-based gene targeting approach or a cross with existing deletion strains followed by random spore analysis (44). All strains in this study are listed in Supplementary Table S1.

### *S. pombe* live-cell imaging

Yeast strains were grown in standard complete YES media (US Biological, cat. Y2060) containing the full complement of yeast amino acids and incubated overnight at 32°C. For PAmCherry-Epe1 strains and Epe1 mutants under the control of the native Epe1 promoter, the seed culture was diluted into the same YES media and incubated at 25°C with shaking to reach an OD_600_ ∼0.5. For strains with the *nmt1*, *nmt41*, or *nmt81* promoter, the seed culture was diluted into EMMC media (Formedium, cat. PMD0402) containing the full complement of yeast amino acids and incubated at 30°C with shaking to reach an OD_600_ ∼0.5. To maintain cells in an exponential phase and eliminate extranuclear vacuole formation, the culture was maintained at OD_600_ ∼0.5 for 2 days with dilutions performed at ∼12-h time intervals (∼24-h time interval for EMM media culture). Cells were pipetted onto a pad of 2% agarose prepared in EMM media and each agarose pad sample was imaged for less than 1 h. *S. pombe* cells were imaged at room temperature with a 100× 1.40 NA oil immersion objective. The fluorescent background was decreased by exposure to 488-nm light (Coherent Sapphire, 377 W/cm^2^ for 20–40 s). A 406-nm laser (Coherent Cube 405–100; 1–5 W/cm^2^) was used for photoactivation (200 ms activation time) and a 561-nm laser (Coherent Sapphire 561–50; 70.7 W/cm^2^) was used for excitation. Images were acquired at 40-ms exposure time per frame. The fluorescence emission was filtered to eliminate the 561-nm excitation source and imaged using a 512 × 512-pixel Photometrics Evolve EMCCD camera.

### Silencing assays

Strains containing the *ura4*+ reporter were grown overnight. Cells were equalized to 1OD/ml then four tenfold dilutions were spotted on nonselective media (EMMC), media lacking uracil (EMM − URA), or media containing 5FOA (EMMC + FOA). 5-flouroorotic acid (5-FOA) was added at a concentration of 1 g/l in EMMC + FOA plates. The plates were incubated at 32°C for 3–4 days before imaging.

### Single-molecule trajectory analysis

Recorded single PAmCherry molecule positions were localized and tracked with SMALL-LABS software (45). A mask of the nucleus of each cell was determined based on autofluorescence outside the nucleus in the 488nm bleaching step. Only the signal within the nucleus mask was analyzed. Single-molecule trajectory datasets were analyzed by a nonparametric Bayesian framework NOBIAS to infer the number of mobility states, the parameter for each state, and the transition between states (29). More than 1000 trajectories for each SPT dataset are put in the framework for robust analysis and to eliminate rare events. Reported parameters for each state are the posterior mean after the number of mobility states stabilizes, and reported uncertainty is the standard deviation from the posterior distribution. Some datasets were also analyzed with two publicly available SPT analysis software DPSP (46) and Spot-On (47). In DPSP analysis, the chosen range of diffusion coefficients was 10^−3^–10 μm^2^/s. In Spot-On analysis, the number of components was fixed to 2 or 3.

### Clustering analysis for the Swi6 distributions

The spatial pattern of each mobility state was investigated using the Ripley's *K* function (48):


\begin{eqnarray*}K\left( r \right) = {{\lambda }^{ - 1}}\mathop \sum \limits_{i = 1}^n \mathop \sum \limits_{i \ne j} \frac{{I\left( {{{r}_{ij}} \,{<}\, r} \right)}}{n}\end{eqnarray*}

where *r* is the search radius, *n* is the number of points in the set, $\lambda$ is the point density, and ${{r}_{ij}}$ is the distance between the *i*th and *j*th point. $I( x )$ is an indicator function. *K*(*r*) is further normalized to a Ripley's *H* function:


\begin{eqnarray*}H\left( r \right) = {{\left( {\frac{{K\left( r \right)}}{\pi }} \right)}^{\frac{1}{2}}} - r\end{eqnarray*}

In this function, $H( r ) = 0$ stands for a random distribution, $H( r ) >0$ means a clustered spatial pattern and $H( r ) \,{<}\, 0$ means a dispersed pattern. In the analysis, the nucleus was approximated as a circle to determine the area and perform edge correction (49). We calculated *H*(*r*) for each cell, and then we calculated an overall *H*(*r*) from the average of all cells weighted by the fits density. To eliminate effects from the spatial correlation between single-molecule steps from the same trajectories, we simulated diffusion trajectories with similar confined area size, average track length, and overall density as experimental trajectories by drawing step lengths from the step size distribution of the corresponding experiment steps. This normalization is reported in previous work (21).

### Reconstructed single-molecule heatmap

For each cell, the nucleus and cell outlines were obtained from the fluorescence image of the nucleus and the phase-contrast image of the cell; these outlines were then approximated by a circle and a rectangle with circular caps, respectively. Every frame was analyzed by SMALL-LABS to identify single molecules, and the position and frame number of each single molecule were saved. To generate the reconstructed single-molecule heatmap for the cell, the pixel intensities after subtraction of the fitted offset in the appropriate diffraction-limited region about each single molecule were summed and the sum of all well-fit molecules was globally normalized.

### Fine-grained chemical rate constant inference

To infer the rate constants of transition for each of these proteins, we used a Bayesian Synthetic Likelihood algorithm similar to the one previously reported for inference of Swi6 rate constants (21). To perform Metropolis Monte Carlo sampling in parameter space, at each step, we began with a set of rate constants and their posterior density. We then proposed a potential new set in one of three ways based on a t-distributed random variable, *T*, with 10 degrees of freedom and the number of protein states *N*: we multiplied a random rate by ${\mathrm{exp}}( {0.02T} )$, we multiplied every rate by ${\mathrm{exp}}( {0.02T/{{N}^2}} )$, or we multiplied a random pair of opposing rates by ${\mathrm{exp}}( {0.01T} )$. We simulated the experimental outcome of the transitions 2000 times for a set of rate constants. For each simulation, we divided the experimental time (0.04 s) into 100-time steps (*δt* = 4 × 10^−4^ s). We assumed that the sampled proteins were partitioned across states according to their equilibrium proportions based on their reported transition matrices from the NOBIAS single-molecule tracking analysis. We sampled from a binomial distribution with transition probability as given above and the number of molecules in a state to determine the number of molecules that transitioned. We used these simulations to calculate a likelihood distribution for the rate constants, multiplied the result by an improper Jeffries prior ($P( {rate} ) \propto 1/rate$), and compared the posterior density to that of the older set of rate constants using the standard Metropolis-Hastings algorithm to decide whether to keep the old set of rates or accept the new one. The posterior mean reaction rates were considered the final posterior rates. We report them with a 95% highest density credible interval. The BSL rate analysis code is available at https://github.com/alimf17/RateConstantsRepo.

### Single-molecule time-lapse imaging

We model the binding of Chp2 and H3K9me or Swi6 to Epe1 as a direct two-component association/dissociation reaction:


\begin{eqnarray*}AB \leftrightharpoons A + B\end{eqnarray*}

The measured residence time of each PAmCherry-Chp2 or PAmCherry-Epe1 molecule is estimated from the lifetime of the stationary fluorescence signal. *k_app_diss_* is acquired by fitting the probability distribution function, *P*, of the measured residence times, *τ_measured_*, to a single exponential decay function:


\begin{eqnarray*}P = {\mathrm{\ exp}}\left( { - {{k}_{app\_diss}} \cdot {{\tau }_{measured}}} \right)\end{eqnarray*}

The measured apparent dissociation rate, *k_app_diss_*, consists of the true dissociation rate, *k_diss_*, and the photobleaching rate of the PAmCherry label, *k_bleaching_*; we separated these contributions by collecting data at multiple delay times to measure the photobleaching rate. For static molecules, we introduced a dark period with each time interval that we kept the integration time, *τ_int_*, the same and introduced different lengths of dark delay times, *τ_delay_*. In this way, the contribution of photobleaching was kept the same for different total time intervals, ${{\tau }_{TL}} = {{\tau }_{int}} + {{\tau }_{delay}}$. We measured the residence time, ${{\tau }_{measured}} = ( {n - 1} ){{\tau }_{TL}}$, by counting the total number of sequential frames, *n*, in which the molecule was detected. Finally, the true dissociation rate, *k_diss_*, was estimated from a linear regression of the two-term relationship (50):


\begin{eqnarray*}{{k}_{app\_diss}}{{\tau }_{TL}} = {{\tau }_{TL}}{{k}_{diss}} + {{\tau }_{int}}{{k}_{bleaching}}\end{eqnarray*}

This linear regression also took the uncertainty of each data point from the exponential fitting into consideration and gave the final fitted slope ${{k}_{diss}}$ and its uncertainty.

### Nucleosome electrophoretic mobility shift assays (EMSAs)

Chp2 proteins were cloned into N-terminal 6XHis-tag containing pET vectors and mutants were generated using ligase-independent cloning (LIC). All Chp2 proteins were purified from BL21(DE3)-RIPL *E. coli* cells. Cells were grown at 37°C to OD 0.5–0.8 in LB media with 100 μg/ml Ampicillin, induced with 0.4 mM Isopropyl-beta-D-thiogalactopyranoside (IPTG), and were grown for 16 h at 18°C. Cells were harvested and resuspended in lysis buffer (1× PBS buffer pH 7.3, 300 mM NaCl, 10% glycerol, 0.1% Igepal CA-630, 1mM PMSF, 1 μg/ml aprotonin, pepstatin A, and leupeptin) and sonicated. Cell debris was removed by centrifugation at 25 000 × g for 35 min. Cell lysates were incubated with HisPur NiNTA resin (ThermoFisher Scientific) at 4°C for at least 2 h. The resin was washed with lysis buffer and protein was eluted (20 mM HEPES pH 7.5, 100 mM KCl, 10% glycerol, and 500 mM imidazole) and the 6× His-tag was cleaved using SUMO protease overnight at 4°C. After cleavage of 6× His-tag, the products were further isolated by anion exchange chromatography using a HiTRAP Q HP column (Cytiva). Proteins were dialyzed into storage buffer (20 mM HEPES, 100 mM KCl, 10% glycerol, 1 mM DTT). Reaction samples were prepared with varying concentrations of Chp2 and 50 nM mononucleosomes (Epicypher, H3K9, and H3K9me3) in binding buffer (20 mM HEPES pH 7.5, 4 mM Tris, 80 mM KCl, 0.1% Igepal CA-630, 0.2 mM EDTA, 2 mM DTT and 10% glycerol). Reactions were incubated at 4°C for 30 min. A 0.5× TBE 6% acrylamide:bis-acrylamide 37.5:1 gel was pre-run at room temperature for at least 1 h at 100 V. Reactions were loaded on the gel and ran under the same conditions for 2.5 h. Gels were post-stained for 30 min with PAGE GelRed DNA stain (Biotium) and imaged using a BioRad GelDoc Imager. The unbound nucleosome band was quantified using ImageJ and binding curves were fit using nonlinear regression (Prism 10).

## Results

### PAmCherry-Chp2 is functional to establish silencing and exhibits normal chromatin occupancy across a wide range of concentrations

**Figure 2.**
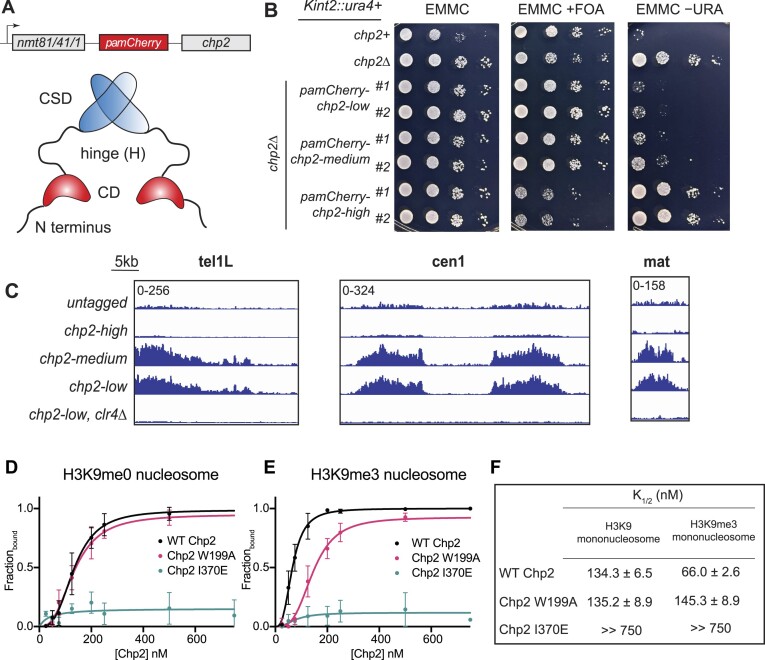
H3K9me and HP1 expression regulate their binding and epigenetic silencing. (**A**) PAmCherry is fused to the N-terminus of Chp2 and expressed ectopically using a series of inducible promoters: *nmt1*, *nmt41*, and *nmt81* (high, medium, and low expression levels, respectively). Bottom: Schematic of the Chp2 domains. CD: chromodomain (H3K9me recognition); H: hinge (nucleic acid binding); CSD: chromoshadow domain (dimerization interface). (**B**) Silencing assay using an *ura4+* reporter inserted at the mat locus (*Kint2::ura4*). 10-fold serial dilutions of cells expressing Chp2 from different *nmt* promoters were plated on EMMC, EMMC + FOA and EMMC − URA plates. All PAmCherry fusion proteins are inserted at the leu1 + locus unless otherwise specified. (**C**) ChIP-seq of PAmCherry-Chp2 showing reads mapped to the *tel1L* locus, *cen1* locus, and mating-type (*mat*) locus for Chp2 expressed from the *nmt1*, *nmt41*, and *nmt81* (high, medium, and low expression levels, respectively). Data shown are reads per million aggregated in 1 kb windows, scaled by 0.01 for ease of visualization (D, E) Concentration dependence curves of quantified electrophoretic mobility shift assays (EMSA) using H3K9me0 **(D)** and H3K9me3 **(E)** mononucleosomes in WT Chp2 (black), Chp2 W199A (pink), and Chp2 I370E (blue). Error bars indicate SD (replicates *N* = 4). (**F**) Table summarizing the apparent binding affinity (*K*_1/2_) and specificity values observed for Chp2-WT, Chp2 W199A and Chp2 I370E.

We previously used single-molecule tracking to identify biophysical mobility states that are associated with distinct biochemical properties of proteins in living cells (21). We had previously determined that Swi6, one of the two HP1 proteins in fission yeast, has four distinct mobility states each of which corresponds to a specific biochemical property in cells (21). Here, we measured the mobility states of the second conserved HP1 protein, Chp2. We labeled the N-terminus of the endogenous copy of Chp2 with photoactivatable mCherry (PAmCherry-Chp2) but were unable to observe an appreciable number of photoactivation events for single-molecule tracking. Instead, we inserted a second copy of an N-terminally labeled PAmCherry-Chp2 under the regulation of thiamine-repressible promoters *nmt1 (high), nmt41 (medium)*, and *nmt81 (low)* (Figure 2A). We performed western blots to quantify PAmCherry-Chp2 expressed using a low (*nmt81*), medium (*nmt41*), and high (*nmt1*) expression promoter (Supplementary Figure S1A). *nmt41*-mediated Chp2 expression produces 5-fold higher protein levels relative to the low expression *nmt81* promoter, whereas *nmt1*-dependent expression leads to ∼100-fold higher protein levels relative to *nmt81* (Supplementary Figure S1A). To evaluate how Chp2 expression affects silencing, we used strains where *ura4+*is inserted at the mating type locus (*Kint2::ura4+*). Chp2 expression using the low-strength (*nmt81*) and medium-strength (*nmt41*) promoters preserved silencing of the *ura4 +*reporter as indicated by successful growth of cells on FOA-containing medium and reduced viability on –URA medium (Figure 2B). We additionally performed chromatin immunoprecipitation of PAmCherry-Chp2 followed by next-generation sequencing. Both the low and medium strength promoters preserved Chp2 occupancy whereas the high expression promoter led to a complete loss of Chp2 chromatin occupancy resembling what we see in *clr4Δ* controls where H3K9me is absent (Figure 2C). We also measured how Chp2 and Swi6 bind differently to nucleosomes *in vitro* using an electrophoretic mobility shift assay (EMSA) (Figure 2D–F). Much like Swi6, wild-type Chp2 binds specifically to H3K9me nucleosomes (Chp2 binds to H3K9me with 2-fold more specificity versus H3K9me0 nucleosomes) (Figure 2F). As expected, a Chp2 chromodomain mutation (W199A) abolishes H3K9me nucleosome binding, leading to the complete loss of specificity and the inability to discriminate between modified and unmodified nucleosomes (Figure 2F). However, in contrast to what we previously observed in the case of the Swi6 dimerization mutant (21), the Chp2 dimerization mutant fails to bind to nucleosomes (Figure 2F). We speculate that this difference is likely because the Swi6 dimerization mutant can still non-specifically bind to nucleic acids, whereas the Chp2 dimerization mutant may not be able to do so to the same extent.

### The *S. pombe* HP1 orthologs, Swi6 and Chp2, exhibit distinct, non-overlapping biophysical states in living cells

Next, we tracked individual PAmCherry-Chp2 molecules expressed from the low expression (*nmt81*) promoter in *S. pombe*. PAmCherry-Chp2 was briefly photoactivated with 405-nm laser light and imaged with 561-nm laser excitation light. We repeated this measurement until all PAmCherry-Chp2 molecules that can be photoactivated were photobleached (Methods). The photoactivation-excitation-imaging cycle was repeated approximately 10–20 times for each cell, and the single molecules were localized and tracked in the recorded fluorescence movies with the SMALL-LABS algorithm (45). We model the motion of Chp2 molecules inside the *S. pombe* nucleus as a diffusive process and thus can assign diffusion coefficients to quantify the different mobility states associated with Chp2. We define a mobility state as a subpopulation of molecules with a distinct diffusion coefficient (*D*). In contrast to the four mobility states that we observed in the case of PAmCherry-Swi6 (a mixture of stationary and mobile molecules), nearly all PAmCherry-Chp2 proteins in *S. pombe* are stationary (Supplementary Figure S1B). Hence, our live cell imaging data reveals a substantially different binding configuration between Swi6 and Chp2.

**Figure 3.**
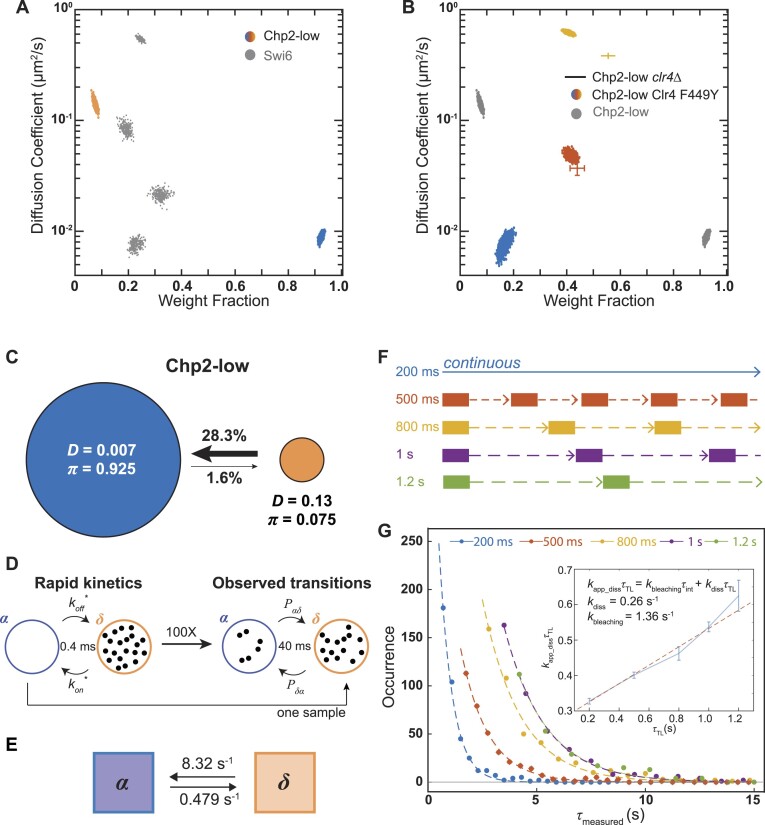
Single-molecule tracking reveals Chp2 dynamics and kinetics. (**A**) NOBIAS identifies two distinct mobility states for PAmCherry-Chp2-low (expressed from the *nmt81* promoter) in WT cells. Each colored point is the average single-molecule diffusion coefficient of PAmCherry-Chp2 molecules in that state sampled from the posterior distribution of NOBIAS inference at a saved iteration after convergence. Grey points are the previously reported PAmCherry-Swi6 single-molecule dynamics (21). (**B**) NOBIAS identifies two distinct mobility states for PAmCherry-Chp2-low in *clr4Δ* cells (cross) and three distinct states for PAmCherry-Chp2-low in Clr4 F449Y cells (colored points). Grey points are the PAmCherry-Chp2-low data (Figure 3A). (**C**) Inferred transition probabilities between the two mobility states of PAmCherry-Chp2-low from single-molecule tracking (Figure 3A). Diffusion coefficients, *D*, in units of μm^2^/s and weight fractions, *π*, are indicated. (**D**) Fine-grained chemical kinetic simulations with Bayesian Synthetic Likelihood algorithm. The reaction on/off rate is proposed and simulated at a 0.4-ms time interval to calculate the likelihood based on transition probabilities from **C** at the 40-ms experimental imaging time interval. (**E)** Inferred rate constants for PAmCherry-Chp2-low. (**F)** Schematic of single-molecule time-lapse imaging. The time-lapse period, ${{\tau }_{TL}}$, is the sum of the 200-ms integration time and the time delay. Five different time delays, ${{\tau }_{TL}}$, were introduced. (**G**) Dwell time distributions for PAmCherry-Chp2-low. The distributions are shown with fits to an exponential decay. Inset: linear fit (red dashed line) of ${{k}_{app\_diss}}{{\tau }_{TL}}$ vs. ${{\tau }_{TL}}$, from which the dissociation rate constant, ${{k}_{diss}}$, and the photobleaching rate constant, ${{k}_{bleaching}}$, are obtained. Errors bars are the standard deviation of the exponential decay fitting.

To investigate any potential heterogeneity in the dynamics within the observed static molecules, we applied NOBIAS, a nonparametric Bayesian framework that can determine the number of mobility states giving rise to a single-molecule tracking dataset (29). We identified two mobility states associated with PAmCherry-Chp2: over 92% of the Chp2 molecules are in the low mobility state with an average diffusion coefficient, *D*_slow, Chp2_ = 0.007 μm^2^/s (Figure 3A) and around 7.5% of Chp2 molecules are in a fast mobility state with *D*_slow,_*Chp2* = 0.13 μm^2^/s. NOBIAS analysis also provides the probability of a molecule transitioning between two mobility states within its trajectory: Chp2 molecules in the fast mobility state are much more likely to transition to the slower state compared with the reverse transition (Figure 3C). These weight fractions and transition probabilities indicate that Chp2 molecules predominantly occupy the slow mobility state and only a very small proportion of Chp2 molecules occupy the fast state. Even when Chp2 expression levels are 5× higher (*nmt41*), over 90% of Chp2 molecules are assigned to the slow mobility state (Supplementary Figure S2B). Notably, *nmt41*-*chp2*+ expression is similar to that of WT Swi6, and yet the two proteins produced very different mobility states in cells. Hence, Chp2 dynamics are very different from those of the second *S. pombe* HP1 protein, Swi6: similar Bayesian analysis using SMAUG found that Swi6 molecules are distributed across four distinct mobility states (21). Overexpression of Chp2 using the *nmt1* promoter (∼100-fold compared to *nmt81*-dependent expression) leads to the emergence of new mobility states, but these states also do not match what we observe in the case of Swi6 (Supplementary Figure S2C). At the highest levels of Chp2 expression, Chp2 outcompetes and promotes the dissociation of Swi6 from heterochromatin (Supplementary Figure S1C), consistent with previous studies (20).

### The loss of the H3K9me binding substrate promotes off-chromatin binding

To determine whether the dominant slow mobility state of Chp2 corresponds to the H3K9me-bound fraction, we deleted Clr4, the sole H3K9 methyltransferase in *S. pombe* (51,52). In a *clr4*Δ background, the slowest PAmCherry-Chp2 mobility state is completely absent (Figure 3B, cross). PAmCherry-Chp2 molecules in *clr4*Δ cells switch over to the fast mobility state consistent with Chp2 proteins moving around the nucleus in an unconstrained manner (weight fraction = 56%, *D_fast_* = 0.36 μm^2^/s). In addition, we observed a new mobility state that we did not previously detect in *clr4+*cells (weight fraction = 44%, *D_int_* = 0.03 μm^2^/s). We hypothesized that this state very likely represents binding to unmethylated chromatin given that the Chp2 chromdomain mutant still binds to unmethylated nucleosomes in our *in vitro* assays (Figure 2D).

To test this model, we performed two orthogonal measurements. First, we expressed a Chp2 chromodomain mutant, PAmCherry-Chp2 W199A which we showed still binds to nucleosomes but lacks specificity (Supplementary Figure S2D). As expected the Chp2 chromodomain mutant leads to a substantial loss of molecules in the low mobility state and a concomitant increase in the fraction of molecules in the intermediate and fast mobility states. We attribute the residual low mobility state (∼10%) to the fact that this mutant retains partial functionality in silencing (53). The intermediate state matches what we observed in *clr4Δ* cells. A second orthogonal measure of Chp2 binding to unmethylated chromatin is how its dynamics change when the only available substrate is H3K9me2 (not me3). H3K9me2 is less preferred as a binding substrate for both Swi6 and Chp2. We tracked single molecules in cells that express a Clr4 mutant (Clr4 F449Y) that is unable to catalyze trimethylation (H3K9me3) (54). We observed a substantial reduction in the slow mobility state consistent with a loss of me3 binding and the appearance of a new intermediate mobility state which once again aligns with the intermediate state we observed in *clr4Δ* cells (Figure 3B). Based on our prior work measuring Swi6 dynamics, the most parsimonious and likely explanation is that this new state represents a chromatin sampling state where Chp2 can potentially bind to unmethylated chromatin, a result that is consistent with our *in vitro* binding assays as well (Figure 2D). To validate the robustness of our results obtained using NOBIAS analysis, we additionally analyzed some of the Chp2 datasets using DPSP and Spot-On, two publicly available SPT analysis software, which showed similar occupancy across different diffusion coefficient ranges (Supplementary Figure S2E–G).

### Chp2 dissociates from H3K9me much more slowly compared to Swi6

The preponderance of the stationary H3K9me-binding state for PAmCherry-Chp2 expressed from the *nmt81* promoter implies that our high-resolution single-molecule tracking measurements may overestimate Chp2 dissociation rates due to photobleaching. This parameter is crucial to determine Chp2 binding kinetics *in vivo* and define the extent to which such measurements correlate with *in vitro* assays. We estimated the dissociation rate using two approaches: (i) single-molecule tracking followed by a Bayesian Synthetic Likelihood (BSL) inference (55). This simulation-based approach has the benefit of not being affected by experimental time-resolution limits; and (ii) single-molecule time-lapse imaging at different time intervals to ensure that photobleaching did not lead to an overestimation of the dissociation rate (50).

To infer the rate constants based on NOBIAS transition matrices, we used a Bayesian Synthetic Likelihood algorithm, which has previously been applied to assess Swi6 dynamics (21,55). BSL methods infer the most justifiable distribution of rate constants to estimate the value and uncertainty of the reaction rate (Figure 3D). We applied the BSL method to analyze the output of the single-molecule tracking analysis and estimated that ${{k}_{diss}} = 0.479\ \pm 0.005\ {{{\mathrm{s}}}^{ - 1}}$ (with the error bounds denoting a 95% credible interval; see Figure 3E). We also experimentally determined Chp2 residence times and dissociation rates using single-molecule time-lapse imaging (Materials and methods). Based on single-molecule time-lapse imaging at five different time intervals (Figure 3F), we calculated a Chp2-H3K9me dissociation rate of ${{k}_{diss}} = 0.260\ \pm 0.018\ {{{\mathrm{s}}}^{ - 1}}$ and an average dwell time of 3.85 s (Figure 3G). In contrast, time-lapse imaging of Swi6 gives ${{k}_{diss}} = 0.454\ \pm 0.051\ {{{\mathrm{s}}}^{ - 1}}$ and an average dwell time of 2.20 s (Supplementary Figure S2H). The *in vivo* time-lapse measurement of Chp2 and Swi6 dissociation rates reveal that Chp2 remains bound to H3K9me for a longer time than Swi6 *in vivo* suggesting that in fact, Chp2 binds to H3K9me chromatin with higher affinity. Our BSL analysis of single-molecule tracking data (21) also confirms that the Chp2 dissociation rate ($0.479\ {{{\mathrm{s}}}^{ - 1}}$) is ∼3-fold lower than that of Swi6 ($1.27\ {{{\mathrm{s}}}^{ - 1}}$). Swi6 and Chp2 both have a chromodomain that is responsible for H3K9me binding specificity (Figure 2A). We asked to what extent Swi6 competes with Chp2 to bind to H3K9me nucleosomes. We imaged PAmCherry-Chp2 in cells lacking Swi6 (*swi6*Δ). The mobility states associated with Chp2, and their occupancies do not change significantly (Supplementary Figure S2A). Furthermore, deleting Swi6 also did not affect the residence time and dissociation rate of PAmCherry-Chp2-low in *swi6*Δcells which revealed a ${{k}_{diss}} = 0.269\ \pm 0.031\ {{{\mathrm{s}}}^{ - 1}}$ and an average dwell time of 3.72 s (Supplementary Figure S2I). The similarity in dissociation rates between PAmCherry-Chp2-low in WT cells and *swi6*Δ cells indicates that although deleting Swi6 perturbs the overall dynamics of Chp2, it does not affect the intrinsic affinity between Chp2 and H3K9me chromatin.

### The anti-silencing factor Epe1 co-localizes with its HP1 binding partner primarily at sites of H3K9 methylation and exhibits limited off-chromatin dynamics

**Figure 4.**
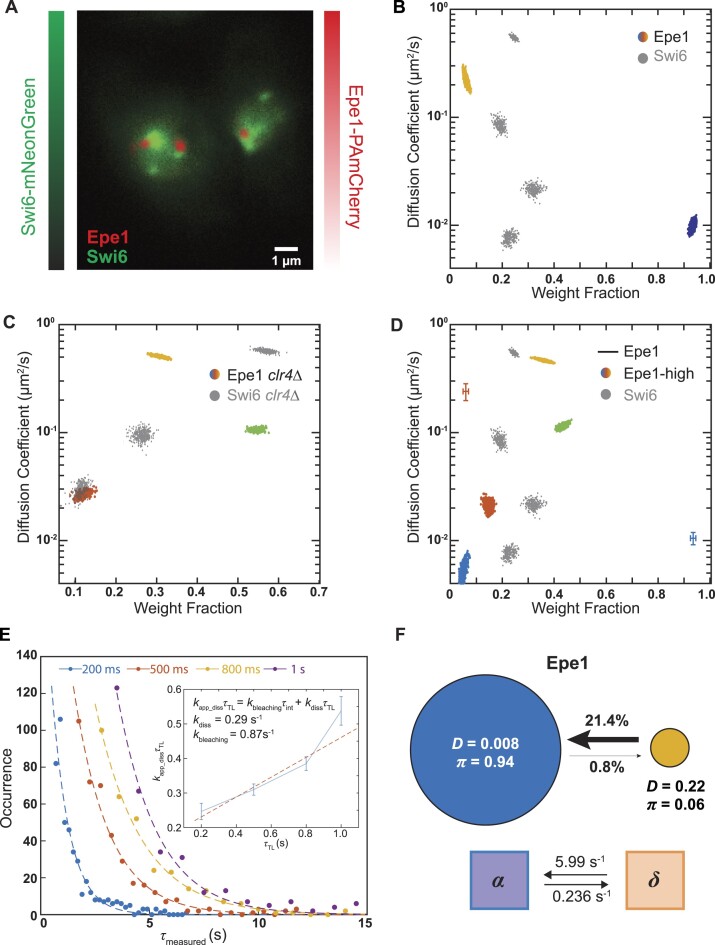
H3K9me regulates HP1-associated protein–protein interactions. (**A**) Two-color imaging of cells expressing mNeonGreen-Swi6 and Epe1-PAmCherry. Swi6 and Epe1 are expressed from their endogenous promoters. Green colorbar: Swi6-mNeonGreen intensities; Red colorbar: reconstructed Epe1-PAmCherry density map. Both color channels are normalized to the maximum pixel intensity. (B–D) NOBIAS identifies distinct mobility states for Epe1-PAmCherry. Each colored point is the average single-molecule diffusion coefficient of PAmCherry-Epe1 molecules in that state sampled from the posterior distribution of NOBIAS inference at a saved iteration after convergence in WT cells (**B**), *clr4Δ* cells (**C**) and highly overexpressed PAmCherry-Epe1-high (from the *nmt1* promoter) (**D**). Grey points are the previously reported PAmCherry-Swi6 single-molecule dynamics in corresponding cells (21). (**E**) Dwell time distributions for Epe1-PAmCherry expressed under its endogenous promoter. The distributions are shown with fits to an exponential decay. Inset: linear fit (red dashed line) of ${{k}_{app\_diss}}{{\tau }_{TL}}$ vs. ${{\tau }_{TL}}$, from which the dissociation rate constant, ${{k}_{diss}}$, and the photobleaching rate constant ${{k}_{bleaching}}$ are obtained. Error bars are from the standard deviation of exponential decay fitting. (**F**) Top: transition probabilities between the two mobility states of Epe1-PAmCherry from (B). Diffusion coefficients, *D*, in units of μm^2^/s and weight fractions, *π*, are indicated. Bottom: Inferred rate constants for Epe1-PAmCherry from the fine-grained chemical kinetic simulation.

The distinct dynamics between Swi6 and Chp2 prompted us to ask how these two HP1 proteins form complexes to facilitate heterochromatin assembly. The putative H3K9me demethylase Epe1 is a major determinant of heterochromatin stability (36,37,56,57). Epe1 directly binds to Swi6 and this interaction is essential for Epe1 recruitment to sites of H3K9me. Deleting Epe1 leads to both unregulated H3K9me spreading and increased epigenetic inheritance (36,38). We labeled Epe1 at its C-terminus with PAmCherry (Epe1-PAmCherry) and confirmed that adding a fluorescent tag does not perturb its silencing properties (Supplementary Figure S3A). To test whether Epe1 molecules successfully localize to heterochromatin sites, we labeled Swi6 with mNeonGreen (mNeonGreen-Swi6) in cells and imaged the emission in the green channel (488-nm excitation) alongside Epe1-PAmCherry in the red channel (561-nm excitation). Overlaying mNeonGreen images with Epe1-PAmCherry super-resolution images indicates that Epe1 foci form at the periphery of Swi6-heterochromatin foci (Figure 4A). This may reflect the role of Epe1 in forming heterochromatin boundaries and being unable to gain access to pericentromeric repeats.

To identify mobility states associated with Epe1, we tracked single Epe1-PAmCherry molecules and inferred the number of mobility states, the diffusion coefficients, and the weight fraction for each Epe1 state. If Epe1 and Swi6 formed complexes away from H3K9me before binding, we would expect to observe four mobility states similar to what we previously observed with Swi6. However, we found that Epe1 has only two mobility states with a predominant slower state (weight fraction, *π_slow_* ∼ 94%, *D_slow, Epe1_* = 0.008 μm^2^/s) (Figure 4B), while only ∼6% of Epe1 are assigned to the faster state (*D_fast, Epe1_* = 0.22 μm^2^/s). The transition probabilities indicate that transitioning from the fast state to the slow state is much more favored than the reverse transition (21% to 0.8%) (Figure 4F). These results suggest that in the presence of H3K9me, Epe1 preferentially remains in the slower H3K9me bound state, presumably through its direct interaction with Swi6. To validate that the recruitment of Epe1 to sites of H3K9me depends on Swi6, we tracked molecules Epe1-PAmCherry molecules in a *swi6*Δ background. As expected, we observed a complete loss of the slow state and the appearance of a new mobility state with a higher diffusion coefficient than the slowest state that we measured in WT cells. In addition, the weight fraction for the fast state, *π_fast_*, increases from 6% in the WT background to over 50% in *swi6*Δ (Supplementary Figure S3D, E).

To determine what role H3K9me might play in promoting complex formation, we tracked single Epe1-PAmCherry molecules in *clr4*Δ cells. As expected, the previously observed Epe1 foci in wild-type cells disappear and Epe1-PAmCherry molecules in *clr4*Δ cells exhibit a diffuse distribution across the nucleus (Supplementary Figure S3F). Also, we observed a complete loss of the slowest state given that neither Swi6 nor Epe1 can localize to sites of heterochromatin in the absence of their cognate H3K9me ligand (Figure 4C). Remarkably, we observed that Epe1 dynamics in *clr4*Δ cells exhibit three mobility states, and the diffusion coefficients of these states align with those of Swi6 (Figure 4C). The alignment in mobility states between Epe1 and Swi6 suggests that the two proteins can directly interact with each other and form off-target complexes in the absence of H3K9me. The transition out of the fast state for Epe1 is 26 times higher than the transition into the fast state (Figure 4F) whereas this ratio decreases to 1.9 in *clr4*Δ cells (Supplementary Figure S3G). These results support the idea that H3K9me chromatin shifts steady-state complex formation between Epe1 and Swi6 towards a chromatin-bound state and suppresses off-chromatin interactions

Next, we performed time-lapse imaging to measure the Epe1 dissociation rate to compare how these rates differ relative to Swi6. We estimated that Epe1 dissociates from sites of heterochromatin formation at a rate that is ${{k}_{diss}} = 0.288\ \pm 0.044\ {{{\mathrm{s}}}^{ - 1}}$ according to single-molecule time-lapse imaging with four-time intervals (Figure 4E). BSL analysis of the single-molecule tracking transition matrix gave a rate of ${{k}_{diss}} = 0.236 \pm 0.003{{s}^{ - 1}}$ (Figure 4F) indicating a mean dwell time of 4.24 ± 0.05 s. These data suggest that Epe1 remains bound to heterochromatin for much longer than Swi6 (2.20 s), on average. These dissociation rate measurements suggest additional contributions beyond just the interaction between Epe1 and Swi6 which promote the persistent and stable association of Epe1 at sites of heterochromatin formation.

Expression levels unquestionably affect the occupancy of proteins across different mobility states. We expressed Epe1 using different strength promoters: *nmt41* and *nmt1*. The medium (*nmt41*) strength promoter resulted in protein expression levels that match WT Swi6, whereas the *nmt1* promoter resulted in 9.2-fold higher expression of protein compared to Swi6 (Supplementary Figure S3B, C). Indeed, under conditions where Epe1 expression is increased, we observed new mobility states relative to what we observed under wild-type Epe1 expression conditions. In the case of expression from *nmt41*, we observed three states: a slow state, a fast state, and an intermediate chromatin sampling state which matches that of Swi6 (Supplementary Figure S3H). In contrast, under *nmt1* expression conditions, we observed four mobility states all of which perfectly match those of Swi6 (Figure 4D). Hence, overexpressing Epe1 to levels in excess of Swi6 achieves the same off-chromatin Epe1 mobility states as deleting Clr4 (*clr4Δ*). As opposed to all of Epe1 being in the chromatin-bound state, overexpressed Epe1 exhibits off-chromatin interactions with Swi6, and these interactions are also found when H3K9me is absent.

### The nucleosome remodeler Mit1 and the histone deacetylase Clr3 also preferentially form complexes only at heterochromatin

Given our observations that Epe1 and Swi6 preferentially form complexes at sites of H3K9me and not off-chromatin, we wanted to determine the extent to which this principle of H3K9me-directed complex assembly can be generalized to other HP1-associated factors. The SHREC complex (a homolog of the Mi2/NuRD complex in humans) consists of a chromatin remodeler Mit1 and histone deacetylase (HDAC) Clr3 both of which are proteins that co-purify with the HP1 protein, Chp2 (Figure 1A). The C-terminus of Chp2 exhibits a high-affinity interaction with the N-terminus of Mit1 and deleting Chp2 (*chp2Δ*) leads to a substantial loss of Mit1 from heterochromatin (41). In contrast, the recruitment of Clr3 depends on both HP1 proteins (Swi6 and Chp2) in addition to exhibiting other HP1-independent binding modes (33,40,58).

**Figure 5.**
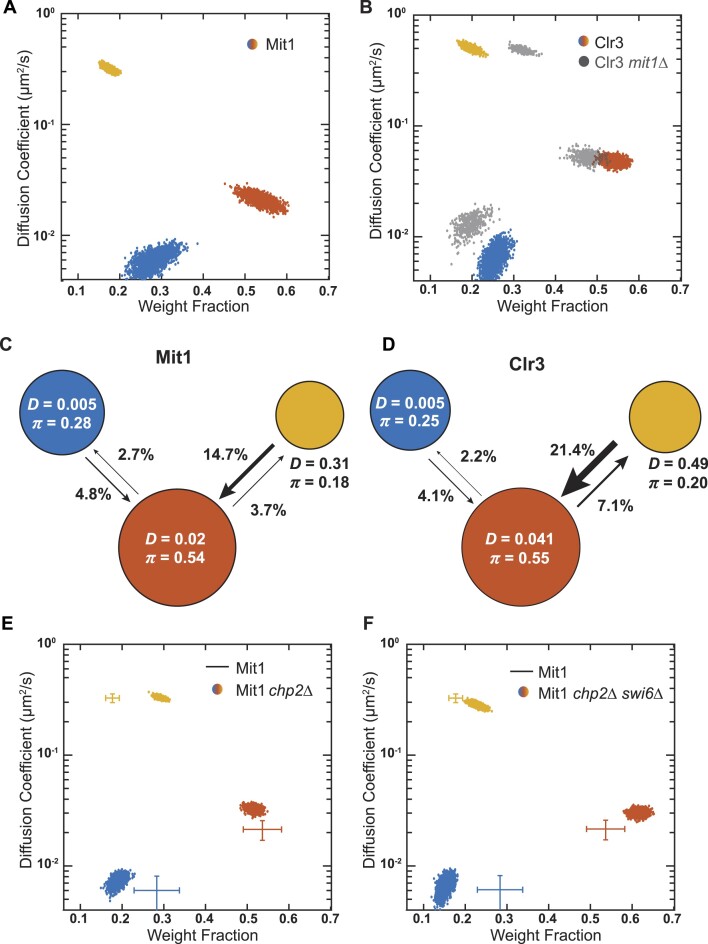
Mit1 and Clr3 preferentially form complexes at heterochromatin. (**A**, **B**) NOBIAS identifies three distinct mobility states for PAmCherry-Mit1 and PAmCherry-Clr3, both expressed from the *nmt81* promoter. Each point is the average single-molecule diffusion coefficient of PAmCherry-Mit1 molecules in WT cells (A) or PAmCherry-Clr3 molecules in WT cells (B, colored points) and in *mit1Δ* cells (B, grey points) that state sampled from the posterior distribution of NOBIAS inference at a saved iteration after convergence. (C, D) Transition probabilities between the three mobility states of PAmCherry-Mit1 (**C**) and PAmCherry-Clr3 (**D**) from NOBIAS. The arrow widths are proportional to the transition probabilities; the transitions with probability below 0.8% are not drawn. Diffusion coefficients, *D*, in units of μm^2^/s and weight fractions, *π*, are indicated. (E, F) NOBIAS identifies three distinct mobility states for PAmCherry-Mit1 molecules from *nmt81 promoter* in *chp2Δ* cells (**E**) and in *chp2Δswi6Δ* cells (**F**). Each point is the average diffusion coefficient and weight fraction of that mobility state sampled from the posterior distribution of NOBIAS inference at a saved iteration after convergence. The cross colors and ranges show data of PAmCherry-Mit1 molecules in WT cells (A).

We previously determined that Chp2 exhibits two distinct mobility states (Figure 3A). Hence, we extended our studies to identify the mobility states associated with its interacting partners, Mit1 and Clr3. We fused PAmCherry to the N-terminus of Mit1 and Clr3 and expressed the two fusion proteins using a thiamine-repressible *nmt81* promoter. We confirmed that both PAmCherry-Clr3 and PAmCherry-Mit1 expression preserve epigenetic silencing of a *ura4+*reporter inserted at the mating-type (*mat*) locus (Supplementary Figure S4A, B). Expressing PAmCherry-Clr3 in *clr3Δ* cells or PAmCherry-Mit1 in *mit1Δ* cells rescues growth in FOA-containing medium and a loss of growth in EMMC−URA medium, consistent with *ura4+*silencing. Our single-molecule tracking data for PAmCherry-Mit1 and PAmCherry-Clr3 reveals that both proteins exhibit three mobility states (Figure 5A, B). The diffusion coefficients for Mit1 and Clr3 only match each other in the slowest states (*D_slow_* = 0.005 μm^2^/s) with comparable weight fractions (28% for Mit1 and 25% for Clr3). The *D_slow_* values for both of these two proteins are again at levels similar to what we have observed in the case of other heterochromatin-associated factors (*D_slow_* of Swi6, Chp2 and Epe1). We analyzed transition probabilities for Mit1 and Clr3 based on our single-molecule tracking data. We noted that Clr3 has a higher probability of transitioning from the fast state to the intermediate state compared with Mit1 (Figure 5C–D). This difference suggests that Mit1 and Clr3 may be recruited to heterochromatin through different mechanisms.

To determine if Mit1 and Clr3 are recruited to heterochromatin independently, we acquired and analyzed single-molecule tracking data for PAmCherry-Mit1 in *clr3Δ* cells and PAmCherry-Clr3 in *mit1Δ* cells. For PAmCherry-Mit1 in *clr3Δ* cells, we still observed three diffusion states with little change to their corresponding diffusion coefficients or weight fractions (Supplementary Figure S4C). In contrast, we observed a slight decrease in the weight fraction of the slow mobility state associated with PAmCherry-Clr3 (from 25.0% to 19.7%) in *mit1Δ* cells (Figure 5B). Hence, these measurements of Mit1 and Clr3 dynamics in different deletion backgrounds show that Mit1 is likely involved in Clr3 binding to heterochromatin.

To quantitatively determine the extent to which Mit1 and Clr3 dynamics depend on the two HP1 proteins, we tracked single molecules in *swi6Δ* and *chp2Δ* cells. We noted that both proteins (PAmCherry-Mit1 and PAmCherry-Clr3) exhibit a decrease in the slow state weight fraction in the absence of Chp2 (*chp2Δ*) compared to WT cells (Figure 5E, Supplementary Figure S4D). In contrast, we observed a similar weight fraction for all three diffusive states of Mit1 and a slight decrease in the slow state weight fraction for Clr3 in the absence of Swi6 (*swi6Δ*) compared to WT (Supplementary Figure S4E,F). Interestingly, Mit1 dynamics in the absence of both HP1 proteins (*swi6Δchp2*Δ*)* had an additive effect leading to a further decrease in the slow state weight fraction observed upon deleting either *swi6Δ* or *chp2Δ* only (Figure 5F). Our results reveal a potential hierarchy in complex formation between Mit1 and the two HP1 proteins, Swi6 and Chp2. Although Mit1 preferentially interacts with Chp2, there is cross-talk between the two HP1 proteins allowing for Swi6 to take over when Chp2 is absent (41).

### H3K9me enables HP1-directed SHREC complex assembly

**Figure 6.**
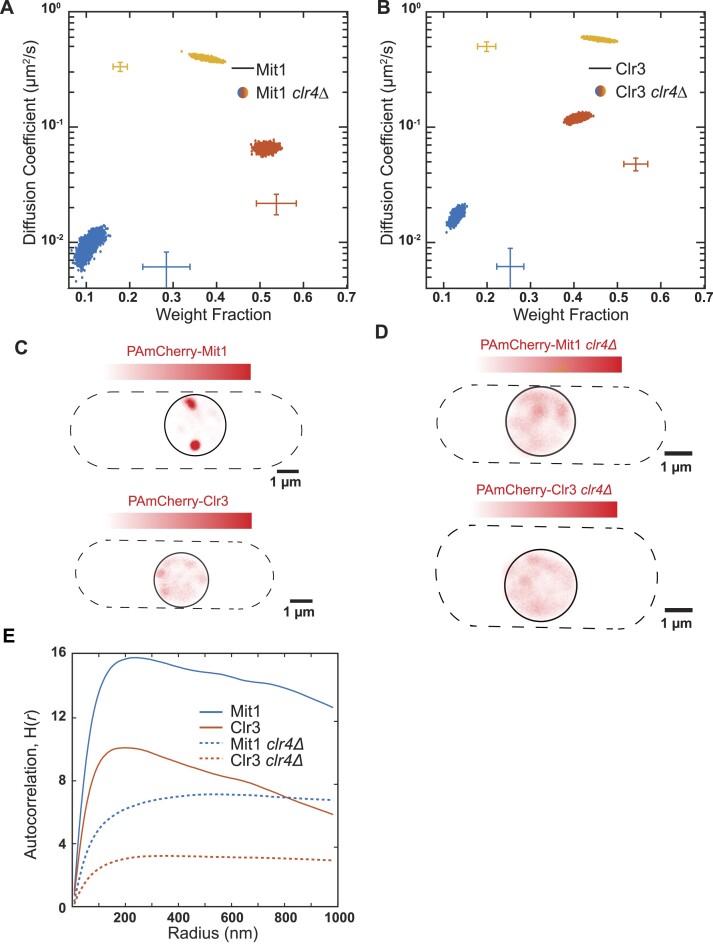
H3K9me enables HP1-directed SHREC complex assembly. (A, B) NOBIAS identifies three distinct mobility states for PAmCherry-Mit1 molecules (**A**) and PAmCherry-Clr3 molecules (**B**) expressed from the *nmt81* promoter in *clr4Δ* cells. Each point is the average diffusion coefficient and weight fraction of that mobility state sampled from the posterior distribution of NOBIAS inference at a saved iteration after convergence. The cross colors and ranges show data for PAmCherry-Mit1 molecules (Figure 5A) or PAmCherry-Clr3 molecules (Figure 5B) from the *nmt81* promoter in WT cells. (C, D) Reconstructed single-molecule density map for PAmCherry-Mit1 (top) and PAmCherry-Clr3 (bottom) expressed from the *nmt81* promoter in WT cells (**C**) and *clr4Δ* cells (**D**). Dashed lines: approximate *S. pombe* cell outlines; solid circles: approximate nucleus borders. (**E**) Ripley's H analysis for steps from all states for Mit1 and Clr3 expressed from the *nmt81* promoter in WT cells and *clr4Δ* cells. Mit1 and Clr3 in *clr4Δ* cells have lower Ripley's *H*(*r*) values than Mit1 and Clr3 in WT cells.

To determine how H3K9me impacts SHREC complex assembly, we tracked single molecules of PAmCherry-Mit1 and PAmCherry-Clr3 in *clr4*Δcells. In *clr4*Δ cells, where H3K9me is absent, the fraction of Mit1 and Clr3 molecules in the slow mobility state (from 28.4% to 10.7% for Mit1 and from 25.5% to 13.0% for Clr3) decrease ∼2-fold. This is accompanied by a corresponding increase in the fast mobility state (from 17.7% to 37.8% for Mit1 and from 20.0% to 46.0% for Clr3) consistent with heterochromatin-associated factors being able to freely diffuse in the nucleus in the absence of H3K9me (Figure 6A,B). However, unlike what we observed in the case of Swi6, Chp2, or Epe1, the slow mobility state is not completely absent for either PAmCherry-Mit1 or PAmCherry-Clr3 in *clr4*Δ cells (∼10%).

Reconstructed single-molecule density heatmaps of Mit1 trajectories in wild-type cells reveal hotspots that match what we typically observed in the case of Chp2, Swi6 and Epe1 whereas Clr3 localizations have a more dispersed pattern (Figure 6C). These results are consistent with Mit1 recruitment being exclusively HP1-dependent whereas Clr3 has additional HP1-independent binding modes (41,58,59). In contrast, reconstructed heatmaps of PAmCherry-Mit1 and PAmCherry-Clr3 in *clr4*Δ where H3K9me is absent completely abolish any clustering (particularly in the case of Mit1) (Figure 6D). We used a Ripley's *H* function to determine the spatial overlap between Mit1 and Clr3 for different mobility states (48). A higher H(*r*) value indicates a higher clustering level at searching radius *r*, which serves as a proxy for whether the two proteins are in the same location. There is little difference between the H functions for the slowest states of Mit1 and Clr3, indicating that the clustering patterns of the slowest Mit1 and Clr3 molecules are likely to arise from their localization at sites of H3K9me (Supplementary Figure S5A). In contrast, Mit1 has a higher *H* function value than Clr3 in the intermediate state across all searching radii, suggesting that the two proteins have different spatial distributions and fail to co-localize when they are unbound from H3K9me (Supplementary Figure S5B). Deleting Clr4 decreases the clustering level for both proteins, but Mit1 still has higher *H*(*r*) than Clr3 in *clr4*Δ cells (Figure 6E). In summary, both the spatial auto-correlation analysis and our single-molecule tracking data for Mit1 and Clr3 suggest that the individual components of the SHREC complex co-localize only at sites of H3K9me and not off-chromatin.

## Discussion

In this work, we captured protein dynamics with super-resolution fluorescence-based imaging and analyzed single-molecule trajectories with Bayesian inference to investigate how heterochromatin-associated factors form complexes with their binding partners in living fission yeast cells. Our observations of the properties of heterochromatin-associated proteins in cells deviate in important and substantive ways from *in vitro* studies. Previous studies have shown that Swi6 binds to nucleosomes with a 3-fold higher affinity than Chp2 (34). In contrast, our data based on the weight fractions of molecules in the H3K9me-dependent slow mobility state, the transition rates of molecules between the free and bound states, and time-lapse imaging to measure *k_off_* demonstrate that the majority of Chp2 molecules are H3K9me-bound and Chp2 binds with higher affinity to H3K9me chromatin. By altering Chp2 protein expression levels, we also reveal how Chp2 binds with high specificity to H3K9me chromatin and the distributions do not change even when Chp2 levels match Swi6 expression levels (Supplementary Figure S2B). Hence, despite the two HP1 proteins having very similar domains, their different amino acid composition, especially within the nucleic acid-binding hinge domain, likely leads to different behaviors in cells. These results might explain why Chp2 is not easily displaced by Swi6 despite the endogenous expression levels of Chp2 protein being 100-fold lower than that of Swi6 (20).

The binding properties of the two HP1 proteins Swi6 and Chp2 serve as an important point of departure for our measurements on heterochromatin complex assembly in living cells. Epe1, a major anti-silencing factor that interacts with Swi6, exhibits only two mobility states. We envisioned two possibilities: (i) If Epe1 binds Swi6 independently of H3K9 methylation, then we would expect the wild-type Epe1 diffusion states to match those of Swi6. (ii) Conversely, if Epe1 only recognizes H3K9me-bound Swi6, we would expect only a slow mobility state and all bound Epe1 molecules to become freely diffusing in a *clr4Δ* background. Our data is most consistent with a model where H3K9me promotes complex formation between Swi6 and Epe1 strongly enough to compete with binary Swi6-Epe1 off-chromatin complexes which we observed only in the case of *clr4Δ* cells or when Epe1 is expressed in large excess over Swi6 (Figure 4C).

These studies are consistent with our earlier observations that the addition of an H3K9me peptide dramatically increased the extent of binding between Epe1 and Swi6 *in vitro* (57). Remarkably, our single-molecule measurements in cells recapitulate the high specificity with which Swi6 and Epe1 form complexes in a heterochromatin-restricted manner, implying that the presence of H3K9me additionally suppresses off-chromatin interactions. The H3K9me chromatin-dependent enhancement also leads to Epe1 having longer dwell times at sites of heterochromatin relative to Swi6 (3.45 s versus 2.22 s). These changes in dwell times could be due to the influence of H3K9me or due to Swi6-dependent compartmentalization via phase separation leading to protein confinement.

We tested whether the principle that H3K9me enhances ternary complex formation could be extended to other proteins such as the chromatin remodeler, Mit1, and the histone deacetylase, Clr3, both of which form complexes with the second *S. pombe* HP1 protein, Chp2. Unlike Chp2, which has only two mobility states, Mit1 and Clr3 exhibit three mobility states. Both Mit1 and Clr3 exhibit mobility states with different diffusion coefficients and spatial autocorrelation functions, except for the slow state which we attribute to an H3K9me-bound fraction. These results suggest that Mit1 and Clr3, which are components of the SHREC complex, co-localize only at sites of H3K9me. Our results are consistent with recent structural work on SHREC complex proteins highlighting the special role that Chp2 plays in recruiting Mit1 to heterochromatin (40,41).

The current study uses single-color tracking to measure the behaviors of proteins that are likely to form a complex; it infers based on mutations and knockouts that the major interaction site is H3K9me-dependent (and conversely, that off-chromatin interactions are not prevalent). We rely on single-color tracking due to technical challenges associated with simultaneous two-color single-molecule tracking. Nevertheless, novel multicolor imaging methods such as proximity-assisted photoactivation (PAPA) present new opportunities to directly track protein interactions and the motions of protein complexes in living cells (60). Unlike our observations of other heterochromatin-associated factors, deleting Clr4 and eliminating all H3K9me does not fully disrupt the slowest Mit1 and Clr3 mobility state. It will be important in the future to determine other orthogonal chromatin and sequence-specific recruitment mechanisms that cause Mit1 and Clr3 to bind in an H3K9me-independent manner.

Chromatin is largely thought to be a scaffold that recruits histone-binding proteins to particular locations in the genome (61). In contrast, our single-molecule measurements of heterochromatin proteins and their binding partners reveal a vital role for H3K9me in enhancing ternary complex formation in living cells. Although the proteins whose properties we measured directly bind to each other and can potentially engage in pairwise binary interactions *in vitro*, we observe little off-heterochromatin co-localization when H3K9me is present. Our results reveal a dramatic shift in the binding states induced by the presence of H3K9me. Most notably, our measurements emphasize the need to explicitly include H3K9me chromatin substrates when formulating models of how heterochromatin-associated factors form complexes both *in vitro* and *in vivo*, given the critical role of chromatin itself in acting as a matchmaker to enhance complex formation. Although the mechanisms of this enhancement are not well understood, it is likely that H3K9me nucleosome binding triggers structural changes in protein conformation. These conformational changes, either in HP1 proteins or their interactors, ultimately lead to ternary complex formation exclusively at sites of H3K9me in living cells (57).

## Supplementary Material

gkae692_Supplemental_File

## Data Availability

The NOBIAS analysis code is available at https://github.com/BiteenMatlab/NOBIAS. The GEO number for the ChIP data in this manuscript is GSE263472. The raw data of single-molecule trajectories are available at https://doi.org/10.5281/zenodo.11544925. The BSL rate analysis code is available at https://github.com/alimf17/RateConstantsRepo and https://zenodo.org/doi/10.5281/zenodo.11544924.

## Notes

Present address: Ziyuan Chen, Department of Molecular and Cell Biology, University of California, Berkeley, CA 94720 USA.

Present address: Saikat Biswas, Pacific Biosciences, Menlo Park, CA 94025, USA.
