# Supplementary material for: Tracking live-cell single-molecule dynamics enables measurements of heterochromatin-associated protein–protein interactions
Source: Nucleic Acids Res. 2024 Aug 15;52(18):10731–46. doi: 10.1093/nar/gkae692 (PMC11472046; doi:10.1093/nar/gkae692)
Supplement: gkae692_Supplemental_File [file gkae692_supplemental_file.pdf]

Figure S1: H3K9me and HP1 expression regulate their binding and epigenetic silencing

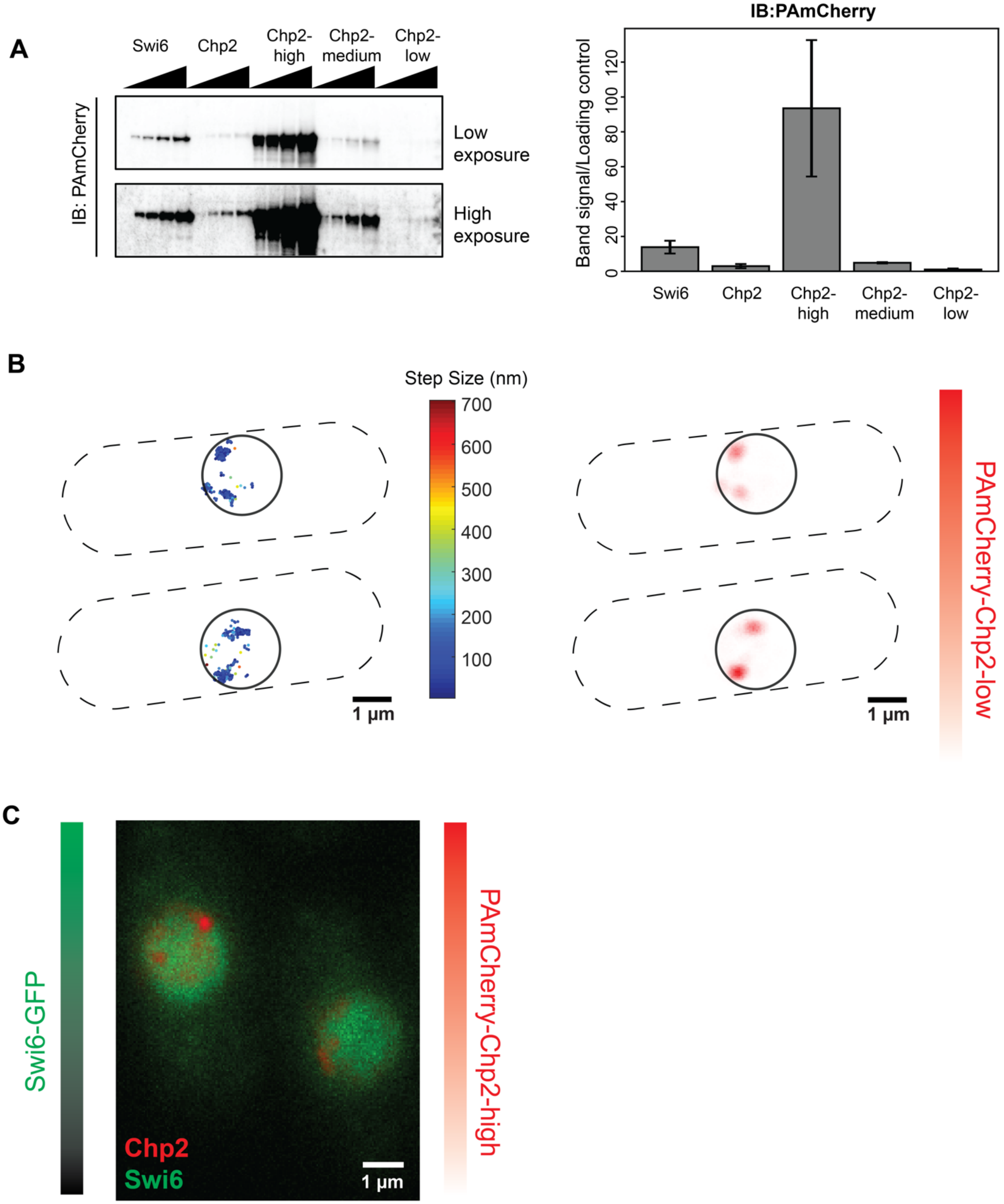

Figure S1. H3K9me and HP1 expression regulate their binding and epigenetic silencing. A: The expression levels of PAmCherry-Chp2 under endogenous (Chp2), *nmt1*(High) *nmt41*(Medium) *nmt81*(Low) promoters and PAmCherry-Swi6 under endogenous promoter, are quantified by western blot against an mCherry antibody. The cross-reactivity of the mCherry antibody can specifically detect PAmCherry protein fusions. All PAmCherry fusion proteins are inserted at the leu1+ locus unless otherwise specified. The relative expression levels of each case are plotted in the bar plot on the right and are normalized to the Chp2 low level. **B**: Left: Single-molecule step size map for PAmCherry-Chp2-Low. Dashed lines: approximate *S. pombe* cell outlines; solid circles: approximate nucleus borders. Right: Reconstructed single-molecule density map of the same cells as the left figure for PAmCherry-Chp2-Low. Dashed lines: approximate *S. pombe* cell outlines; solid circles: approximate nucleus borders. **C**: Two-color imaging of cells with Swi6-GFP expressed from the endogenous promoter and PAmCherry-Chp2-High. Green colorbar: Swi6-GFP intensities; red colorbar: reconstructed PAmCherry-Chp2 density map. Both color channels are normalized to the maximum pixel intensity.

Figure S2: Single-molecule tracking reveals Chp2 dynamics and kinetics

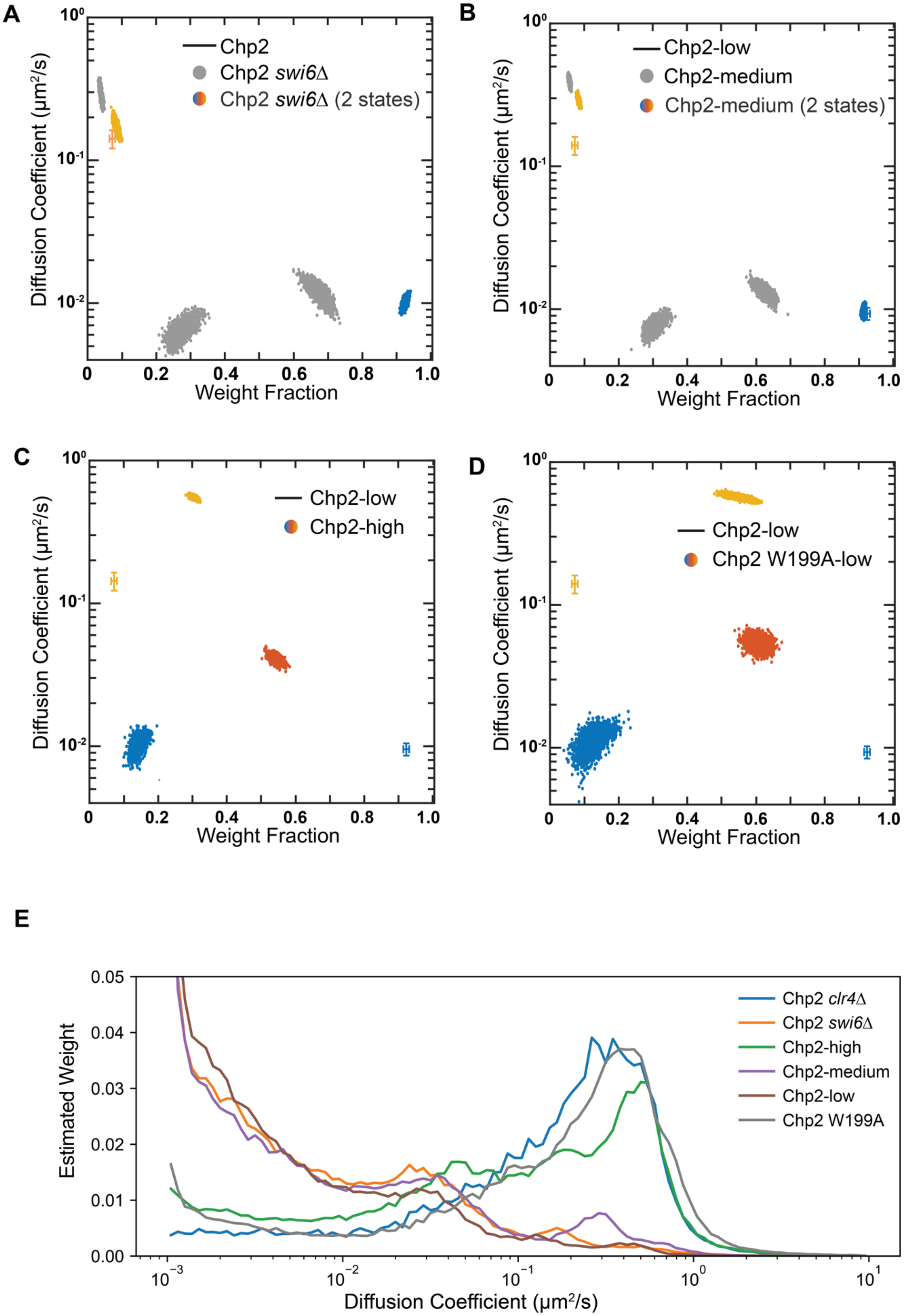

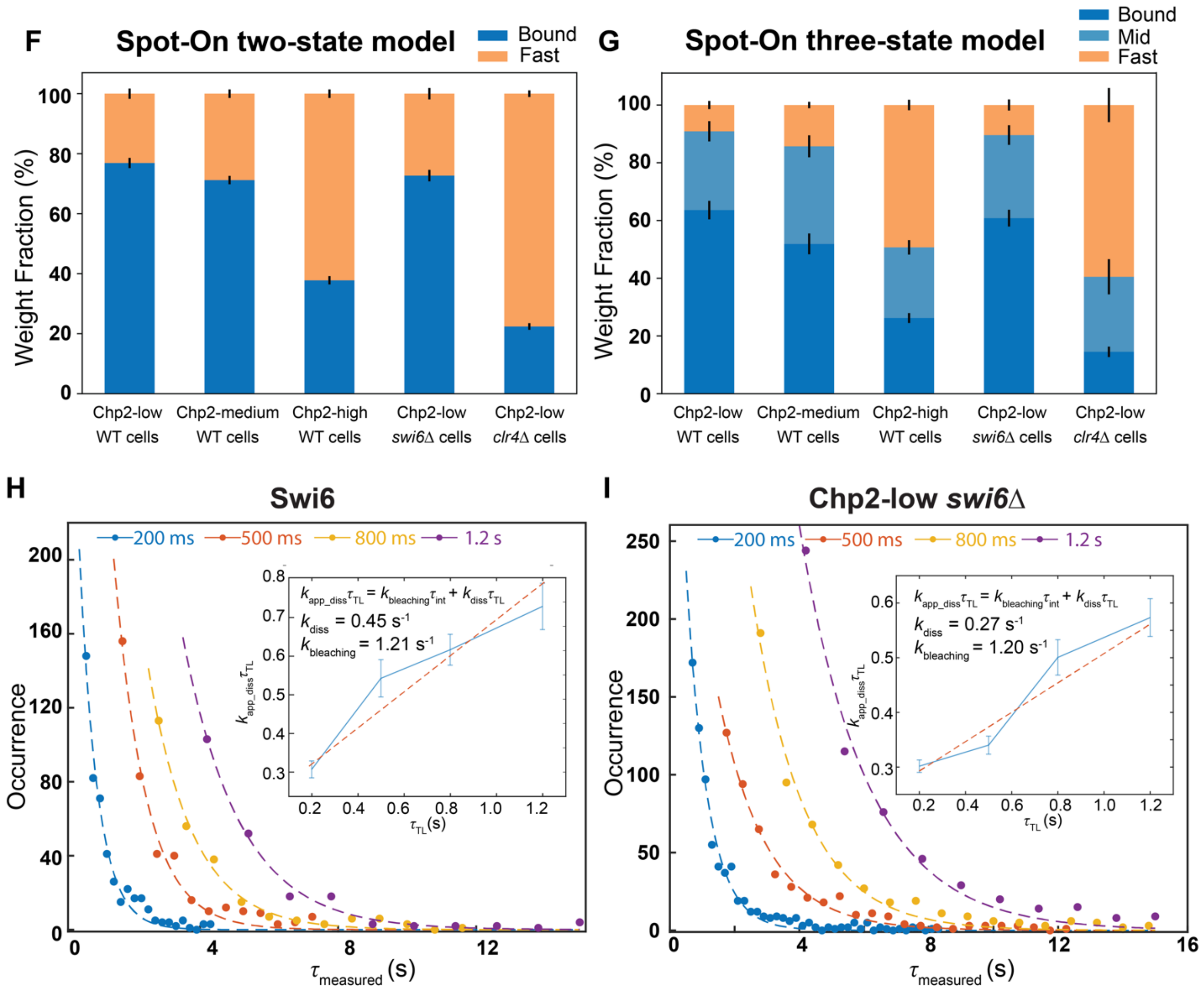

Figure S2: Single-molecule tracking reveals Chp2 dynamics and kinetics. A-B: NOBIAS identifies distinct mobility states for PAmCherry-Chp2-low ((*nmt81* promoter)) in *swi6Δ* cells and PAmCherry-Chp2-medium (*nmt41* promoter). Each point is the average single-molecule diffusion coefficient of molecules in that state sampled from the posterior distribution of NOBIAS inference at a saved iteration after convergence. The colored crosses show the data for PAmCherry-Chp2-low in WT cells (Figure **3A**). Due to overspliting in the NOBIAS algorithm, both the 3-state results (grey points) and the results for constraining the algorithm to 2 states (colored points) are shown. **C-D:** NOBIAS identifies distinct mobility states for PAmCherry-Chp2-high with *nmt1 promoter* and PAmCherry-Chp2-W199A-low mutant with *nmt81* promoter. Each colored point is the average single-molecule diffusion coefficient of molecules in that state sampled from the posterior distribution of NOBIAS inference at a saved iteration after convergence. The colored crosses show the data for PAmCherry-Chp2-low in WT cells (Figure **3A**). **E**: Posterior distribution of diffusion coefficients of single-molecule trajectory datasets inferred from DPSP analysis (46). **F-G**: Weight fractions of each mobility state for various PAmCherry-Chp2 single-molecule trajectory datasets inferred from Spot-On analysis (47) with a two-state model (**F**) and a three-state model (**G**). **H-I**: Dwell time distributions for PAmCherry-Swi6 expressed under its endogenous promoter (**H**) and PAmCherry-Chp2-low in *swi6Δ* cells(**I**).

The distributions are shown with fits to an exponential decay. Insert: linear fit (red dashed line) of $k_{app\_diss}\tau_{TL}$ versus τ_{TL}, from which the dissociation rate constant k_{diss} and the photobleaching rate constant $k_{bleaching}$ are obtained. Errors bars are the standard deviation of the exponential decay fitting.

Figure S3: H3K9me regulates HP1-associated protein-protein interactions

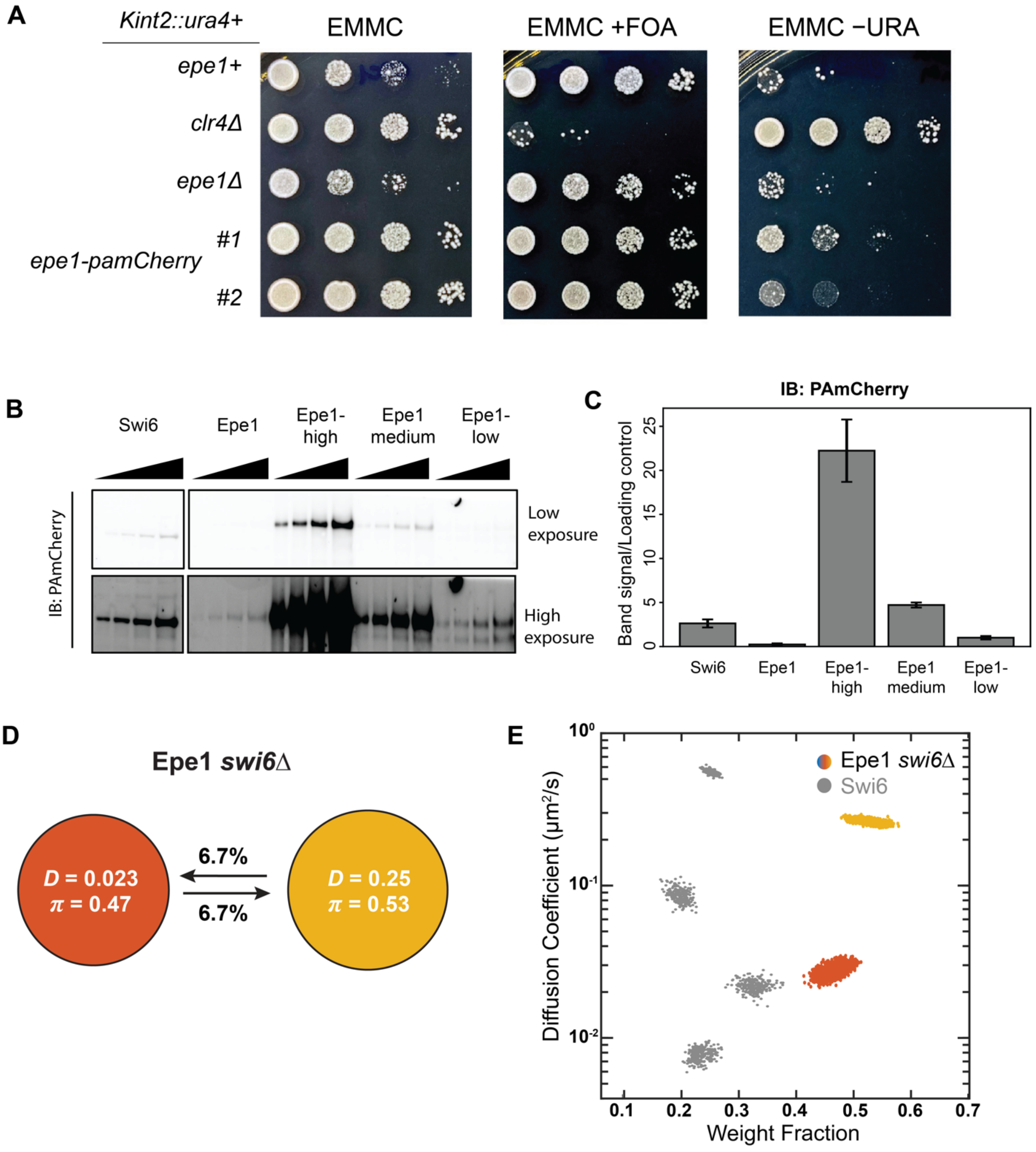

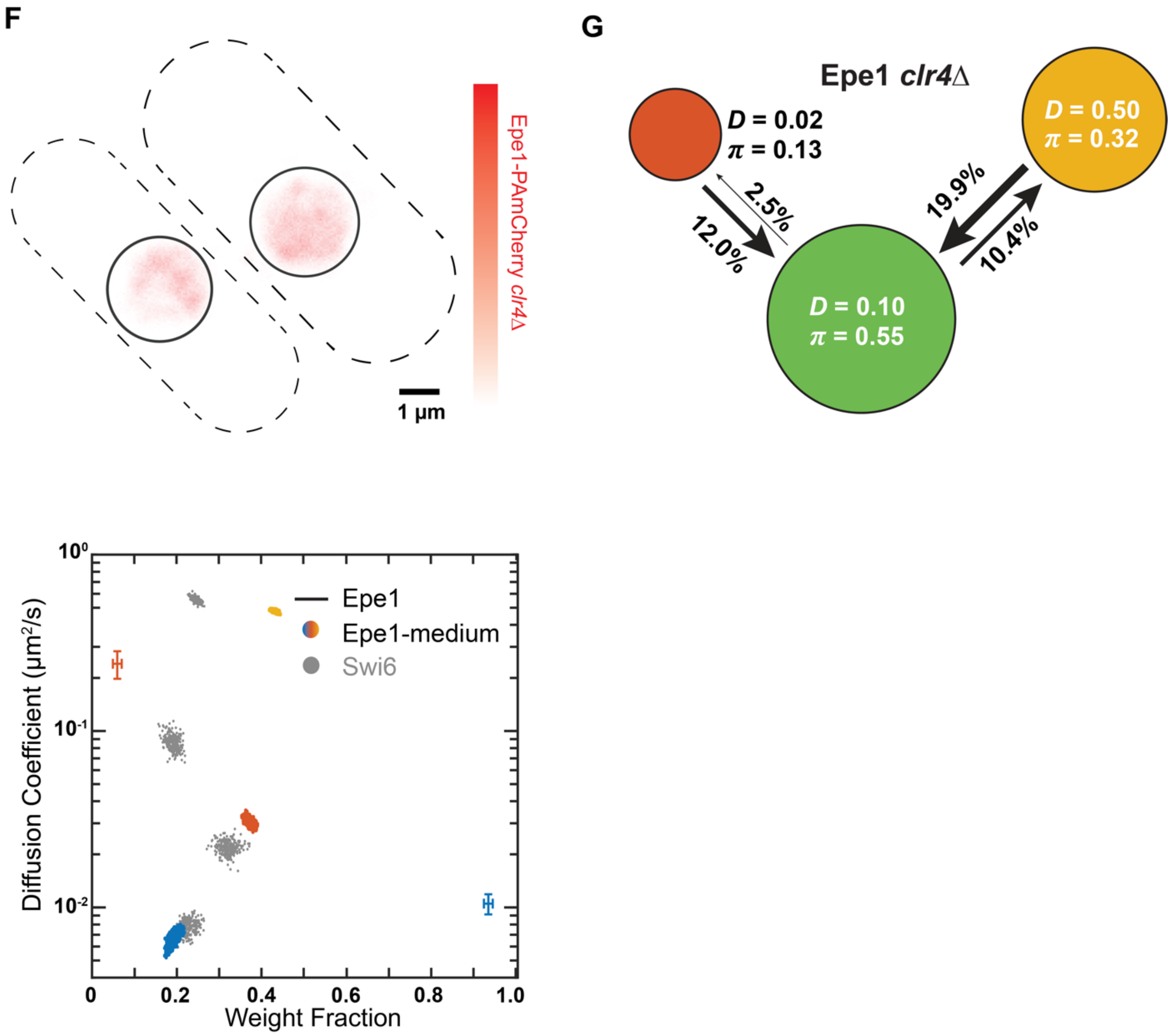

Figure S3: H3K9me regulates HP1-associated protein-protein interactions. A: Silencing assay using an *ura4*+ reporter inserted at the mat locus (*Kint2::ura4).* 10-fold serial dilutions of cells expressing Epe1 from its endogenous locus were plated on EMMC, EMMC +FOA, and EMMC-URA plates. **B**: The expression levels of Epe1-PAmCherry under endogenous, *nmt1*(high) *nmt41*(medium) *nmt81*(low) promoters and PAmCherry-Swi6 under endogenous promoter, are quantified by western blot. All PAmCherry fusion proteins are inserted at the leu1+ locus unless otherwise specified. **C:** The relative expression levels of each case in **B** and are normalized to the Epe1-low level. **D, G**: Inferred probabilities between the mobility states of Epe1-PAmCherry under endogenous promoter in *swi6Δ* cells (**D**) and in *clr4Δ* cells (**G**) from single-molecule tracking with NOBIAS. The arrow sizes are proportional to the transition probabilities. **E, H:** NOBIAS identifies distinct mobility states for PAmCherry-Epe1 under endogenous promoter in *swi6Δ* cells and PAmCherry-Epe1-medium under *nmt41 promoter* in WT cells. Each colored point is the average single-molecule diffusion coefficient of molecules in that state sampled from the posterior distribution of NOBIAS inference at a saved iteration after convergence. The colored crosses show the data for PAmCherry-Epe1 in WT cells (Figure **4B**).

Grey points are the previously reported PAmCherry-Swi6 single-molecule dynamics in corresponding cells (27). **F**: Reconstructed single-molecule density map of the same cells as the left figure for PAmCherry-Epe1 under endogenous promoter in *clr4Δ* cells. Dashed lines: approximate *S. pombe* cell outlines; solid circles: approximate nucleus borders.

Figure S4: Mit1 and Clr3 preferentially form complexes at heterochromatin

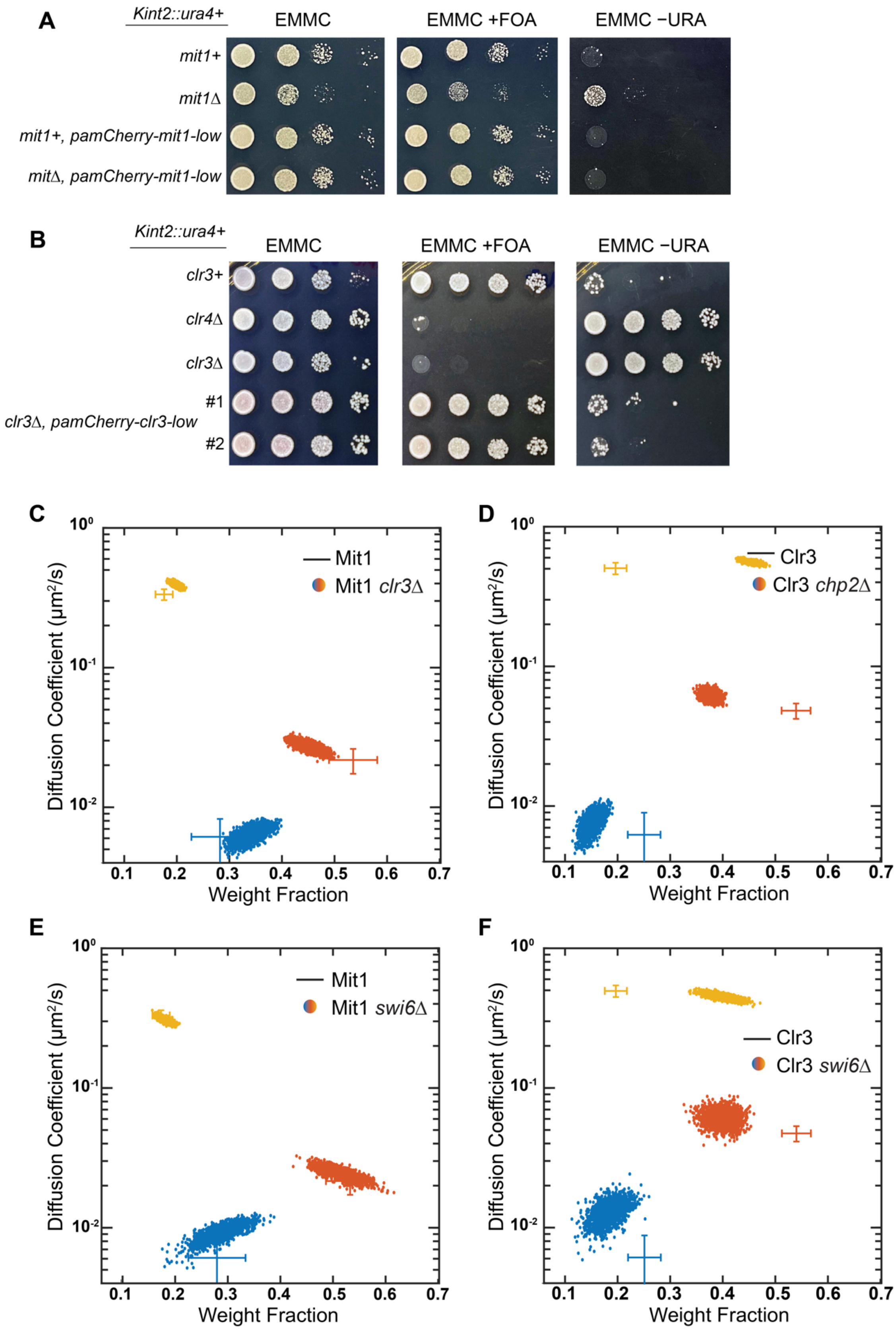

Figure S4. Mit1 and Clr3 preferentially form complexes at heterochromatin. A-B: Silencing assay using an *ura4+* reporter inserted at the mat locus (*Kint2::ura4).* 10-fold serial dilutions of cells expressing Mit1 (**A**) and Clr3 (**B**) from ectopic *nmt81* promoter were plated on EMMC, EMMC +FOA, and EMMC-URA plates. All PAmCherry fusion proteins are inserted at the leu1+ locus unless otherwise specified. **C, E:** NOBIAS identifies distinct mobility states for PAmCherry-Mit1 under *nmt81* promoter in *clr3Δ* cells and *swi6Δ* cells. Each colored point is the average single-molecule diffusion coefficient of molecules in that state sampled from the posterior distribution of NOBIAS inference at a saved iteration after convergence. The colored crosses show the data for PAmCherry-Mit1 under the *nmt81* promoter in WT cells (Figure **5A**). **D, F:** NOBIAS identifies distinct mobility states for PAmCherry-Clr3 from *nmt81* promoter in *chp2Δ* cells and *swi6Δ* cells. Each colored point is the average single-molecule diffusion coefficient of molecules in that state sampled from the posterior distribution of NOBIAS inference at a saved iteration after convergence. The colored crosses show the data for PAmCherry-Clr3 from *nmt81* promoter in WT cells (Figure **5B**).

Figure S5: H3K9me enables HP1-directed SHREC complex assembly

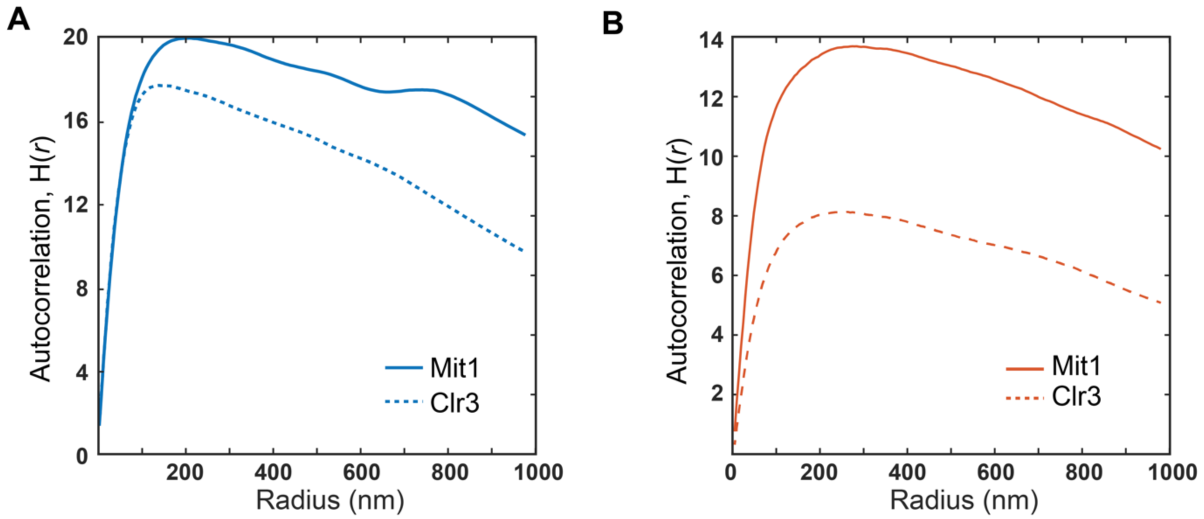

Figure S5: H3K9me enables HP1-directed SHREC complex assembly. A: The slow state of Mit1 (solid line) has a similar Ripley's H(*r*) value to the slow state of Clr3 (dashed line), indicating that both proteins are clustered in slow states (both proteins are expressed from the *nmt81* promoter in WT cells). Each autocorrelation plot is normalized to randomly simulated trajectories from the same state (Methods). **B:** The intermediate state of Mit1 (solid line) has a higher Ripley's H(*r*) than the intermediate state of Clr3 (dashed line). Each autocorrelation plot is normalized with randomly simulated trajectories from the same state (Methods).

Table S1: List of strains used.

Strain no.	*Strain genotype*	*Source*	*Related to*
KR2659	*h90 ade6-M210 his2 leu1-32 ura4DS/E kint2::ura4+ chp2Δ::kan*	Moazed lab	Figure 2B
KR2771	*h90 ade6-M210 his2 leu1-32 ura4DS/E kint2::ura4+ chp2Δ::kan, leu1+:nmt41-pamcherry-chp2-ADHt #1*	This study	Figure 2B
KR2772	*h90 ade6-M210 his2 leu1-32 ura4DS/E kint2::ura4+ chp2Δ::kan, leu1+:nmt41- pamcherry-chp2-ADHt #2*	This study	Figure 2B
KR2773	*h90 ade6-M210 his2 leu1-32 ura4DS/E kint2::ura4+ chp2Δ::kan, leu1+:nmt1- pamcherry-chp2-ADHt #1*	This study	Figure 2B
KR2774	*h90 ade6-M210 his2 leu1-32 ura4DS/E kint2::ura4+ chp2Δ::kan, leu1+:nmt1- pamcherry-chp2-ADHt #2*	This study	Figure 2B
KR2775	*h90 ade6-M210 his2 leu1-32 ura4DS/E kint2::ura4+ chp2Δ::kan, leu1+:nmt81- pamcherry-chp2-ADHt #1*	This study	Figure 2B
KR2776	*h90 ade6-M210 his2 leu1-32 ura4DS/E kint2::ura4+ chp2Δ::kan, leu1+:nmt81- pamcherry-chp2-ADHt #2*	This study	Figure 2B
KR2598	*h90 ade6-M216 leu1+ nmt81-pamcherry-chp2-ura4-D18*	This study	Figure 3A
KR2616	*h90 leu1+:nmt81-pamcherry-chp2, clr4::kanMX6#1*	This study	Figure 3B
KR3103	*h90 ade6-M216 leu1-32 ura4-D18 clr4::myc-clr4F449Y natMX6 leu1+:nmt81-pamcherry-chp2 #3*	This study	Figure 3B
KR2620	*h90 leu1+:nmt81-pamcherry-chp2, swi6::natMX6 #2*	This study	Figure S2A
KR2662	*h90 ade6-M216 leu1+ nmt1-pamcherry-chp2-ura4-D18 #1*	This study	Figure S2B
KR2664	*h90 ade6-M216 leu1+ nmt41-pamcherry-chp2-ura4-D18 #1*	This study	Figure S2C
KR3169	*h90 ade6-M210 his2 leu1-32 ura4DS/E Mint2::ura4+ chp2D::kan leu1+:pamcherry-chp2-W199A #1*	This study	Figure S2D
KR1176	*h90 leu1+:mneongreen-swi6-kanMX6-epe1-pamcherry hphMX6*	This study	Figure 4AB
KR2710	*h90 epe1-pamcherry-hphMX6, clr4Δ::kanMX6#1*	This study	Figure 4C
KR3128	*h90 epe1-pamcherry-hphMX6, Kint2::ura4+ #2*	This study	Figure S3A
KR3129	*h90 epe1-pamcherry-hphMX6, Kint2::ura4+ #4*	This study	Figure S3A
KR2601	*h90 epe1-pamcherry-hphMX6, swi6Δ::natMX6 #1*	This study	Figure S3DE
KR3221	*h90 ade6-M216 leu1-32 ura4-D18 leu2:leu+ nmt1-epe1-pamcherry #1*	This study	Figure 4D
KR3223	*h90 ade6-M216 leu1-32 ura4-D18 leu2:leu+ nmt41-epe1-pamcherry #1*	This study	Figure S3H
KR3225	*h90 ade6-M216 leu1-32 ura4-D18 leu2:leu+ nmt81-epe1-pamcherry #1*	This study	Figure S3B
KR2322	*h90 ade6-M216 leu1+ nmt81-pamcherry-mit1 ura4-D18 #10*	This study	Figure 5A
KR2597	*h90 ade6-M216 leu1+ nmt81-pamcherry-clr3-ura4-D18*	This study	Figure 5B
KR2432	*h90 leu1-32 his2- ura4 DS/E ade6-M210 Kint2::ura4+, leu1+:nmt81-pamcherry-mit1#1*	This study	Figure S4A

KR2434	*h90 leu1-32 his2- ura4 DS/E ade6-M210 Kint2::ura4+, mit1Δ, leu1+:nmt81- pamcherry-mit1 #1*	This study	Figure S4A
KR3289	*h90 leu1+: nmt81-pamcherry-clr3, his2- ura4 DS/E ade6-M210 Kint2::ura4+ clr3D::kanMX6 #1*	This study	Figure S4B
KR3290	*h90 leu1+: nmt81-pamcherry-clr3, his2- ura4 DS/E ade6-M210 Kint2::ura4+ clr3D::kanMX6 #2*	This study	Figure S4B
KR2407	*h90 leu1-32 ura4DS/E ade6-M210? otr1R::ura4 mit1-pamcherry-hphMX6 leu1+:nmt81-pamcherry-mit1 clr4Δ::kanMX6 #4*	This study	Figure 6A
KR2607	*h90 ade6-M216 leu1+ nmt81-pamcherry-clr3-ura4-D18 clr4Δ::kanMX6 #1*	This study	Figure 6B
KR2411	*h90 leu1-32 ura4DS/E ade6-M210? otr1R::ura4? mit1-pamcherry-hphMX6 leu1+:nmt81-pamcherry-mit1 chp2Δ::kanMX6 #9*	This study	Figure 5E
KR2563	*h90 leu1-32 ura4DS/E ade6-M210? otr1R::ura4? mit1-pamcherry-hphMX6 leu1+:nmt81- pamcherry-mit1 chp2Δ::kanMX6 swi6D::natMX6 #1*	This study	Figure 5F
KR2404	*h90 leu1-32 ura4DS/E ade6-M210? otr1R::ura4? mit1-pamcherry-hphMX6 leu1+:nmt81-pamcherry-mit1 swi6Δ::natMx6 #1*	This study	FigureS4E
KR2604	*h90 ade6-M216 leu1+:nmt81-pamcherry-clr3-ura4-D18 chp2Δ::kanMX #1*	This study	FigureS4D
KR2611	*h90 ade6-M216 leu1+:nmt81-pamcherry-clr3-ura4-D18 swi6Δ::natMX6 #2*	This study	FigureS4F
KR2412	*h90 leu1-32 ura4DS/E ade6-M210? otr1R::ura4? mit1-pamcherry-hphMX6 leu1+:nmt81- pamcherry-mit1 clr3Δ::kanmx6 #1*	This study	FigureS4C
KR2615	*h90 ade6-M216 leu1+:nmt81-pamcherry-clr3 ura4-D18 mit1Δ #7*	This study	Figure 5B
